# NG2 glia-derived GABA release tunes inhibitory synapses and contributes to stress-induced anxiety

**DOI:** 10.1038/s41467-021-25956-y

**Published:** 2021-09-30

**Authors:** Xiao Zhang, Yao Liu, Xiaoqi Hong, Xia Li, Charles K. Meshul, Cynthia Moore, Yabing Yang, Yanfei Han, Wei-Guang Li, Xin Qi, Huifang Lou, Shumin Duan, Tian-Le Xu, Xiaoping Tong

**Affiliations:** 1 Center for Brain Science, Shanghai Children’s Medical Center, School of Medicine, Shanghai Jiao Tong University, Shanghai, China; 2 Department of Anatomy and Physiology, Shanghai Jiao Tong University School of Medicine, Shanghai, China; 3 Institute of Special Environmental Medicine, Co-innovation Center of Neuroregeneration, Nantong University, Nantong, Jiangsu China; 4 Research Services, VA Medical Center, Portland, OR USA; 5 Department of Behavioral Neuroscience and Pathology, Oregon Health & Science University, Portland, OR USA; 6 Department of Neurobiology, Key Laboratory of Medical Neurobiology of Ministry of Health of China, Zhejiang University School of Medicine, Hangzhou, Zhejiang China; 7 Shanghai Research Center for Brain Science and Brain-Inspired Intelligence, Shanghai, China

**Keywords:** Glial biology, Molecular neuroscience

## Abstract

NG2 glia, also known as oligodendrocyte precursor cells (OPCs), play an important role in proliferation and give rise to myelinating oligodendrocytes during early brain development. In contrast to other glial cell types, the most intriguing aspect of NG2 glia is their ability to directly sense synaptic inputs from neurons. However, whether this synaptic interaction is bidirectional or unidirectional, or its physiological relevance has not yet been clarified. Here, we report that NG2 glia form synaptic complexes with hippocampal interneurons and that selective photostimulation of NG2 glia (expressing channelrhodopsin-2) functionally drives GABA release and enhances inhibitory synaptic transmission onto proximal interneurons in a microcircuit. The mechanism involves GAD67 biosynthesis and VAMP-2 containing vesicular exocytosis. Further, behavioral assays demonstrate that NG2 glia photoactivation triggers anxiety-like behavior in vivo and contributes to chronic social defeat stress.

## Abstract (web summary)

Nerve/glial antigen 2 (NG2) glia can sense synaptic inputs from neurons. Here, the authors show NG2 glia form functional GABAergic synapses by regulating inhibitory synaptic transmission onto adjacent hippocampal interneurons, and activation of NG2 glia induces anxiety-like behaviour in a mouse model of chronic social defeat stress.

## Introduction

NG2 glia, also known as oligodendrocyte precursor cells (OPCs), were first identified in the 1980s and displayed self-renewal functionality as multipotent stem cells by providing myelinating oligodendrocytes during early brain development^1–3^. These progenitors are highly abundant at birth and ubiquitously distributed throughout the adult brain. In the ~30 years of research progress trying to understand the role of NG2 glia, it has been gradually revealed that they play important and diverse functions in the central nervous system (CNS) in both healthy and pathological conditions^4–12^. However, how NG2 glia interact with neurons and affect neural networks at the synaptic or ultrastructural level remains unclear. It is now widely accepted that astroglia participate in neuronal circuitries as tripartite synapses and contribute to synaptic information processing in health and disease^13–18^. The most intriguing facet of NG2 glia is the fact that they represent the only type of glial cells that receives direct synaptic inputs from both glutamatergic and GABAergic neurons and exhibits neuronal-like long-term potentiation (LTP) at excitatory synapses^4,6,9^. NG2 glia respond to vesicular or non-vesicular synaptic inputs and detect quantal neurotransmitter release from proximal neurons or unmyelinated axons^10,19–23^, suggesting that NG2 glia could share properties previously thought to be restricted to neurons and have a greater impact on neuronal networks^3,8,20,21^. Therefore, it is imperative to gain a full understanding of whether these synaptic interactions between neurons and NG2 glia are bidirectional or unidirectional, as previous research has been mostly focused on unitary neuron-to-NG2 glia synapses. Here, using optogenetic activation of NG2 glia, combined with NG2 glia selective genetic, transcriptomic, electrophysiological, total internal reflection fluorescence (TIRF) imaging, immuno-electron microscopy (EM), and behavioral analysis approaches, we discovered that NG2 glia form NG2 glia-to-neuron synaptic complexes and that selective photostimulation of NG2 glia expressing channelrhodopsin-2 (ChR2) functionally drives GABA release, thereby enhancing inhibitory synaptic transmission onto adjacent interneurons in a microcircuit. We also provide evidence that NG2 glia relaying GABA signals involves GAD67 biosynthesis and VAMP-2 containing vesicular exocytosis. Further behavioral studies suggest that NG2 glia activation induces anxiety-like behavior in a chronic social defeat stress (CSDS) mouse model.

## Results

### Optogenetic activation of NG2 glia in Pdgfrα-creER^TM^; ChR2(H134R)-eYFP transgenic mouse

**Fig. 1:**
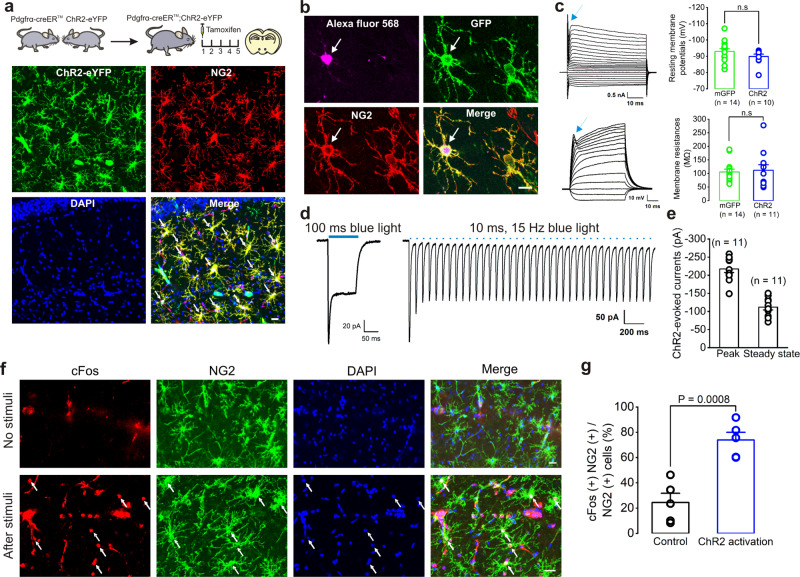
Specific activation of NG2 glia by optogenetic stimulation. **a** Cartoon illustrating the procedure for generating transgenic mouse line by breeding Pdgfrα-creER^TM^ with ChR2(H134R)-eYFP (Ai32) mice to induce ChR2 expression in NG2 glia after consecutive 5-day tamoxifen injections. The immunohistochemistry images below show the ChR2-eYFP labeling (in green) is specifically expressed in NG2 antibody-labeled NG2 glia (in red) in adult hippocampus from Pdgfrα-creER^TM^;ChR2(H134R)-eYFP mice at postnatal 4–6 weeks. The colocalization is indicated by arrows. Scale bar, 20 μm. **b** Representative images show a whole-cell patched NG2 glia expressing ChR2-eYFP loaded with Alexa Fluor 568 in the patch pipette in the stratum radiatum region of the adult hippocampus at P30 and identified with post-immunostaining of NG2 antibody. The colocalization is indicated by arrows. Scale bar, 20 μm. **c** Whole-cell voltage-clamp and current-clamp recordings from NG2 glia showing a typical sodium channel current depolarization but not generating action potentials as the arrows indicate (voltage steps: –160–70 mV, 10 mV/step; current steps: −200–1200 pA, 100 pA/step). The summary bar graphs show there is no change of the cell membrane properties after ChR2 expression in NG2 glia compared with its mGFP control (resting membrane potentials for mGFP, −93.0 ± 1.7 mV, *n* = 14 cells; ChR2, −89.9 ± 1.4 mV, *n* = 10 cells, Mann–Whitney test, *P* = 0.1721; membrane input resistances for mGFP, 105.5 ± 10.3 MΩ, *n* = 14 cells; ChR2, 112.1 ± 20.8 MΩ, *n* = 11 cells, two-tailed unpaired *t* test, *P* = 0.7645). **d** Representative traces show that a 100 ms blue light pulse or 10 ms 15 Hz light pulses stimulation at 5 mW/mm^2^ illumination intensity induces one or a series of non-degrading photocurrents in NG2 glia from Pdgfrα-creER^TM^; ChR2 mouse hippocampus at postnatal 4–6 weeks. **e** Bar graph summarizing the peak and steady-state ChR2-evoked photocurrents in NG2 glia. *n* = 11 cells. **f** Representative images of cFos staining in NG2 glia before and after ChR2 photoactivation. The arrows indicate a significant increase of cFos-positive NG2 glia after blue light stimuli in acute hippocampal slices at postnatal 4–6 weeks. Scale bars, 20 μm. **g** Summary graph shows the percentage increase of cFos-positive NG2 cells in a whole population of NG2 glia after photostimulation. No blue light stimuli: 24.5 ± 7.23%; After blue light stimuli: 73.8 ± 6.14%, *n* = 5 mice, two-tailed unpaired *t* test, *P* = 0.0008. Data are presented as mean values ± SEM and the error bar represents SEM.

The algal protein channelrhodopsin-2 (ChR2), a rapidly gated light-sensitive cation channel, has been broadly used as a genetic tool to deliver into mammalian neurons and probe neural signals in the brain circuitry^24–26^. To gain further insight into whether NG2 glia can exhibit a presynaptic-like function, we took advantage of crossing transgenic mouse lines by breeding Pdgfrα-creER^TM^ with ChR2(H134R)-eYFP (Ai32) or ROSA26-mGFP reporter mice^11,27,28^. Consistent with previous studies, we confirmed that Pdgfrα is a cell-specific marker for NG2 glia and immunohistochemistry from Pdgfrα-creER^TM^;ChR2(H134R)-eYFP mice showed a 94.69 ± 0.89% (*n* = 1524 cells from 12 mice) colocalization between YFP-labeled cells and NG2 antibody and a 100% (*n* = 746 cells from 7 mice) colocalization between YFP-labeled cells and Olig2 (Oligodendrocyte lineage cell marker). Although there was a 6.93 ± 1.62% (*n* = 284 cells from 3 mice) colocalization between YFP-labeled cells and mature oligodendrocyte marker CC1, they were not colocalized with the neuronal marker NeuN nor with the astrocytic marker GFAP in the hippocampus, suggesting that Pdgfrα-creER^TM^;ChR2(H134R)-eYFP mouse strain is specific for the oligodendrocyte lineage and mostly labels NG2 glia (Fig. 1a and Supplementary Fig. 1). After confirming the specific labeling of NG2 glia in a Pdgfrα-creER^TM^ recombinase mouse strain, we performed a series of functional tests on adult NG2 glia with ChR2 expression after tamoxifen induction. First, we identified ChR2-eYFP-labeled cells as NG2 glia by post-immunostaining with NG2 antibody after the cell was loaded with Alexa Fluor 568 from the patch pipette (Fig. 1b). Consistent with previous studies^4,6^, these NG2 glia showed a typical small sodium channel current but were not able to generate action potentials (APs) after cell depolarization, and the basic membrane properties of ChR2-expressing NG2 glia were not affected when compared with mGFP controls (Fig. 1c, *n* = 14 and 10 cells for mGFP-expressing and ChR2-expressing NG2 glia, respectively). Second, when given a 100 ms blue light pulse or 10 ms 15 Hz light pulse stimulation at 5 mW/mm^2^ illumination intensity, one photocurrent or a series of non-degrading photocurrents were reliably induced in NG2 glia from Pdgfrα-creER^TM^;ChR2-eYFP mouse hippocampus (Fig. 1d, e, *n* = 11 cells). By using different intensities of blue light stimulation, NG2 glia exhibit an intensity-response effect on ChR2-evoked photocurrent as well as membrane depolarization (Supplementary Fig. 2). As 5 mW/mm^2^ illumination intensity induces a measurable increase of ChR2-evoked current of −103.01 ± 20.48 pA (*n* = 8 cells) and 4.71 ± 0.81 mV (*n* = 10 cells) membrane depolarization at steady state in NG2 glia, we set 5–10 mW/mm^2^ illumination intensity as an appropriate protocol for optogenetic stimulation in most of in vitro and in vivo experiments^25,29,30^. Third, we assessed immediate-early gene (cFos) expression in NG2 glia following optical stimulation. The cFos staining showed a threefold increase in NG2 glia after photostimulation in acute hippocampal slices, indicating that NG2 glia were indeed activated after the blue light stimuli were applied to ChR2-expressing cells (Fig. 1f, g, *n* = 5 mice).

### NG2 glia photoactivation selectively enhances phasic and tonic inhibition onto hippocampal interneurons

**Fig. 2:**
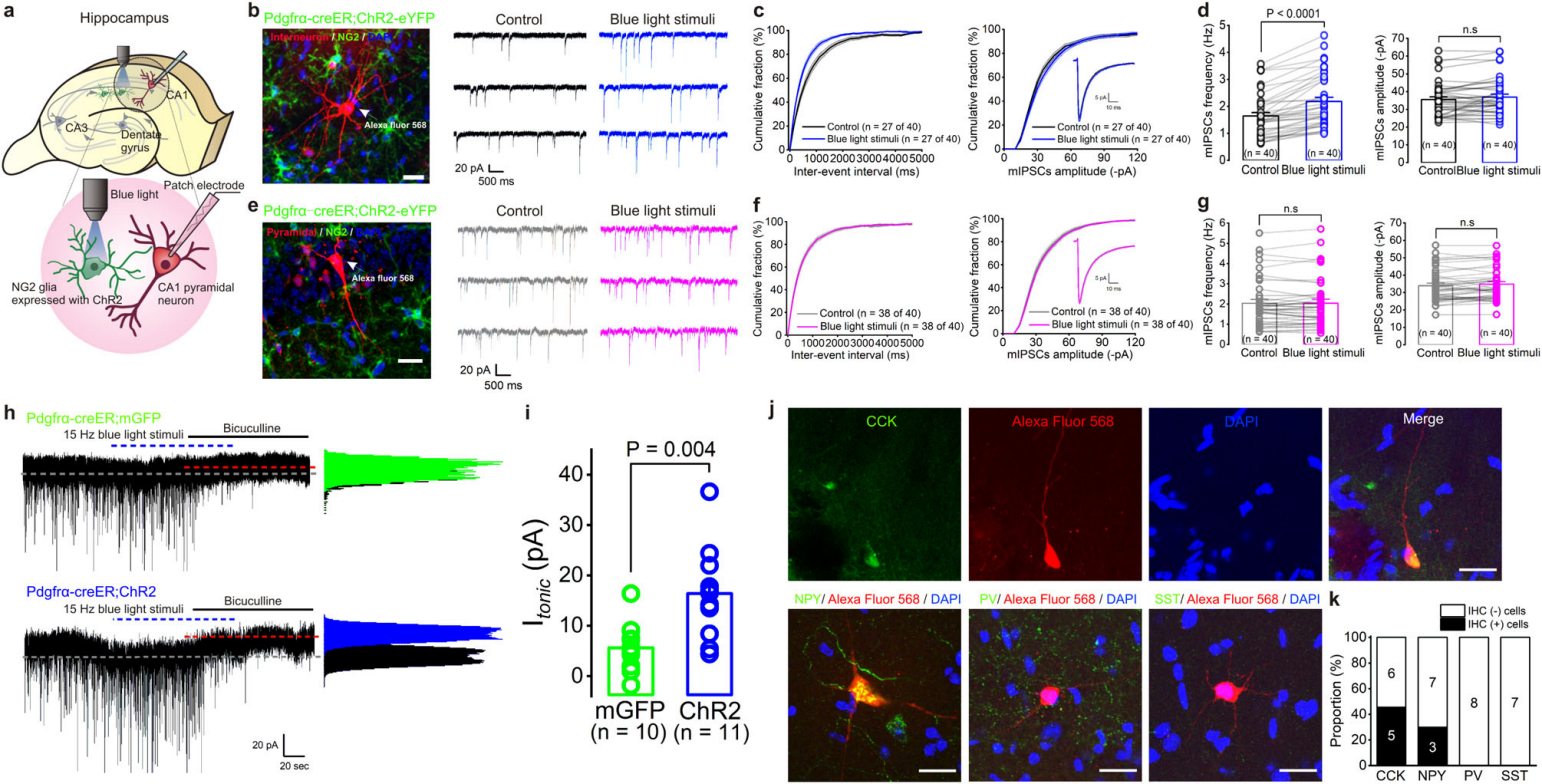
Selective enhancement of phasic and tonic inhibition onto interneurons by NG2 glia photostimulation. **a** Cartoon illustrating the anatomic location of cells for electrophysiology and photostimulations in CA1 region of the hippocampus at postnatal 4–6 weeks. **b** Representative traces (right panel) showing an increase of frequency but not the amplitude of mIPSCs from a whole-cell patched interneuron loaded with Alexa Fluor 568 in the patch pipette (left panel) after NG2 glia photostimulation in CA1 region. Scale bar, 20 μm. **c** and **d** Summary graphs for cumulative distributions of mIPSCs frequency and amplitude (**c**) and mean frequency and amplitude of mIPSCs (**d**) from recorded interneurons. *n* = 40 cells, two-tailed Wilcoxon-matched pairs test, *P* values as indicated. Note that the average of GABA_A_Rs kinetics in the inset shows no change after NG2 glia photostimulation. **e** Representative traces (right panel) showing no change of frequency and amplitude of mIPSCs of a whole-cell patched pyramidal neuron loaded with Alexa Fluor 568 in the patch pipette (left panel) after NG2 glia photostimulation in CA1 pyramidal cell layer. Scale bar, 20 μm. **f** and **g** Summary graphs for cumulative distributions of mIPSCs frequency and amplitude (**f**) and mean frequency and amplitude of mIPSCs (**g**) in recorded pyramidal neurons. *n* = 40 cells, two-tailed paired *t* test, n.s. indicates not significant. Note that the average of GABA_A_Rs kinetics in the inset shows no change after NG2 glia photostimulation. **h** Representative traces showing an increase of GABA_A_R-mediated tonic current in an interneuron after NG2 glia photoactivation (lower panel) from Pdgfrα-creER^TM^;ChR2-eYFP mouse compared with its basal tonic inhibition in an interneuron (upper panel) from Pdgfrα-creER^TM^;mGFP mouse. **i** Summary graph showing a significant enhancement of tonic GABA current in ChR2 compared with its mGFP control after blue light stimulation. Tonic GABA currents: 5.63 ± 1.59 pA, *n* = 10 cells in mGFP control vs. 16.38 ± 2.78 pA, *n* = 11 cells in ChR2, two-tailed unpaired *t* test, *P* = 0.004. **j** Representative images show post-immunostaining CCK-, NPY-, PV-, or SST- antibody with Alexa Fluor 568-loaded interneurons, which exhibited an enhanced frequency of mIPSCs after NG2 glia photoactivation. Scale bars, 20 μm. **k** The summary graph shows the proportion of each CCK+, NPY+, PV+, or SST+ interneurons from a total of 36 responsive interneurons recorded. Data are presented as mean values ± SEM and the error bar represents SEM.

Synaptic strength, along with the neuron’s intrinsic excitability, is a plastic property of cells that can be regulated/modulated when a micro-environmental change alters a neuronal circuit. To directly address the functional consequences of selective NG2 glia photoactivation on neuronal synaptic activity, miniature inhibitory postsynaptic currents (mIPSCs) and miniature excitatory postsynaptic currents (mEPSCs) were recorded using whole-cell patch-clamp separately from both hippocampal interneurons and CA1 pyramidal neurons in Pdgfrα-creER^TM^; ChR2-eYFP transgenic mice, in the presence of tetrodotoxin (TTX) to block the AP-evoked synaptic transmission. As the cartoon and images presented in Fig. 2a, b, and e show, one interneuron and one pyramidal neuron loaded with a fluorescent dye Alexa Fluor 568 in the patch pipette were easily identified in the hippocampal CA1 region based on their anatomical locations, distinct morphologies, as well as firing properties^31,32^. In the presence of 20 µM DNQX, 50 µM AP5 and 1 µM TTX to block glutamatergic as well as AP-evoked synaptic transmission, we found that the frequency of mIPSCs of interneurons was significantly enhanced in 27 out of 40 randomly recorded cells without affecting the amplitude nor the dynamics of mIPSCs after NG2 glia photostimulation (15 Hz, 90 sec) (Fig. 2b, c, Kolmogorov–Smirnov two-sample test). The average frequency and amplitude of mIPSCs in a total of 40 interneurons were: 1.64 ± 0.13 Hz pre- vs. 2.18 ± 0.16 Hz post-NG2 glia photoactivation (*P* < 0.0001, *n* = 40 cells, two-tailed Wilcoxon-matched pairs test); 35.50 ± 1.55 (-pA) pre- vs. 36.92 ± 1.63 (-pA) post-NG2 glia photoactivation (*P* = 0.1206, *n* = 40 cells, two-tailed Wilcoxon-matched pairs test, Fig. 2d and Supplementary Fig. 3), respectively. However, neither frequency nor amplitude of mIPSCs showed significant changes in 95% of recorded pyramidal neurons after NG2 glia photoactivation (38 out of 40 cells, Fig. 2e, f, Kolmogorov–Smirnov two-sample test). The average frequency and amplitude of mIPSCs in a total of 40 pyramidal neurons were: 2.04 ± 0.19 Hz pre- vs. 2.04 ± 0.19 Hz post-NG2 glia photoactivation (*P* = 0.9839, *n* = 40 cells, two-tailed Wilcoxon-matched pairs test); 33.94 ± 1.48 (-pA) pre- vs. 34.89 ± 1.47 (-pA) post-NG2 glia photoactivation (*P* = 0.0748, *n* = 40 cells, two-tailed paired *t* test, Fig. 2g) before and after NG2 glia photoactivation, respectively. A recent study showed that optogenetic depolarization of mature oligodendrocytes facilitates hippocampal pyramidal neurons’ excitatory synaptic responses^33^. To exclude that the synaptic effect might be caused by a small population of ChR2-expressing oligodendrocytes (6.93 ± 1.62% from 3 mice) shown in our immunohistochemistry analysis (Supplementary Fig. 1), we recorded mEPSCs using whole-cell patch-clamp in both pyramidal neurons and interneurons of hippocampal CA1 region. In the presence of 20 µM bicuculline and 1 µM TTX, we found that neither interneurons’ nor pyramidal neurons’ mEPSCs were altered after NG2 glia photoactivation (Supplementary Fig. 4, *n* = 12 and 11 for interneurons and pyramidal neurons, respectively), indicating no or non-measurable effects from photostimulated oligodendrocytes.

GABA_A_Rs mediate two distinct types of inhibition in the brain: phasic and tonic. Phasic inhibition, as represented by IPSCs, arises due to the release of GABA from presynaptic neurons and tonic inhibition arises due to the persistent activation of extrasynaptic GABA_A_Rs in response to ambient and synaptic GABA spillover^16,34,35^. To determine how NG2 glia photoactivation affects interneurons’ inhibitory synaptic activity, we applied 20 μM bicuculline in the presence of DNQX and AP5 to evoke tonic GABA currents in interneurons and found that a 2.9-fold increase of tonic inhibition occurred after NG2 glia photoactivation (Fig. 2h, i), which indicates a GABA release/spillover in the synaptic cleft and a presynaptic regulation from NG2 glia to interneurons. In addition, to further identify which subtype of interneuron in hippocampal CA1 region that NG2 glia could potentially target, we combined electrophysiological and histological methods by post-immunostaining cholecystokinin- (CCK-), neuropeptide Y- (NPY-), parvalbumin- (PV-), or somatostatin- (SST-) antibody with Alexa Fluor 568-loaded interneurons, which exhibited an enhanced frequency of mIPSCs after NG2 glia photoactivation. The percentage of CCK and NPY positive GABAergic neurons constituted 45% and 30% from 11 and 10 responding interneurons, respectively (Fig. 2j, k). Concordant with previous findings, we did not detect high numbers of PV or SST-positive GABAergic neurons in the hippocampal CA1 region including stratum radiatum, lacunosum, and moleculare layer^36,37^. However, in the dentate gyrus (DG), we found an enhanced frequency of mIPSCs of interneurons after NG2 glia photoactivation in the hilus, which is highly enriched with SST- and PV-interneurons (Supplementary Fig. 5, *n* = 12 cells from 4 mice)^36,37^. Considering the similar effect on proximal interneurons in both hippocampal CA1 and DG region, we next used systemic diphtheria toxin (DT) administration to ablate NG2 glia in Pdgfrα-CreER^TM^/ChR2-eYFP/iDTR mice (Supplementary Fig. 6a, b). Indeed, the enhancement of mIPSCs frequency in hippocampal interneurons was diminished (Supplementary Fig. 6c, *P* > 0.05, two-tailed paired *t* test, *n* = 10 cells from 3 mice). Therefore, we conclude that NG2 glia could provide a general synaptic regulation of hippocampal interneurons in a microcircuit.

### NG2 glia form pre- and postsynaptic structures with adjacent hippocampal neurons

**Fig. 3:**
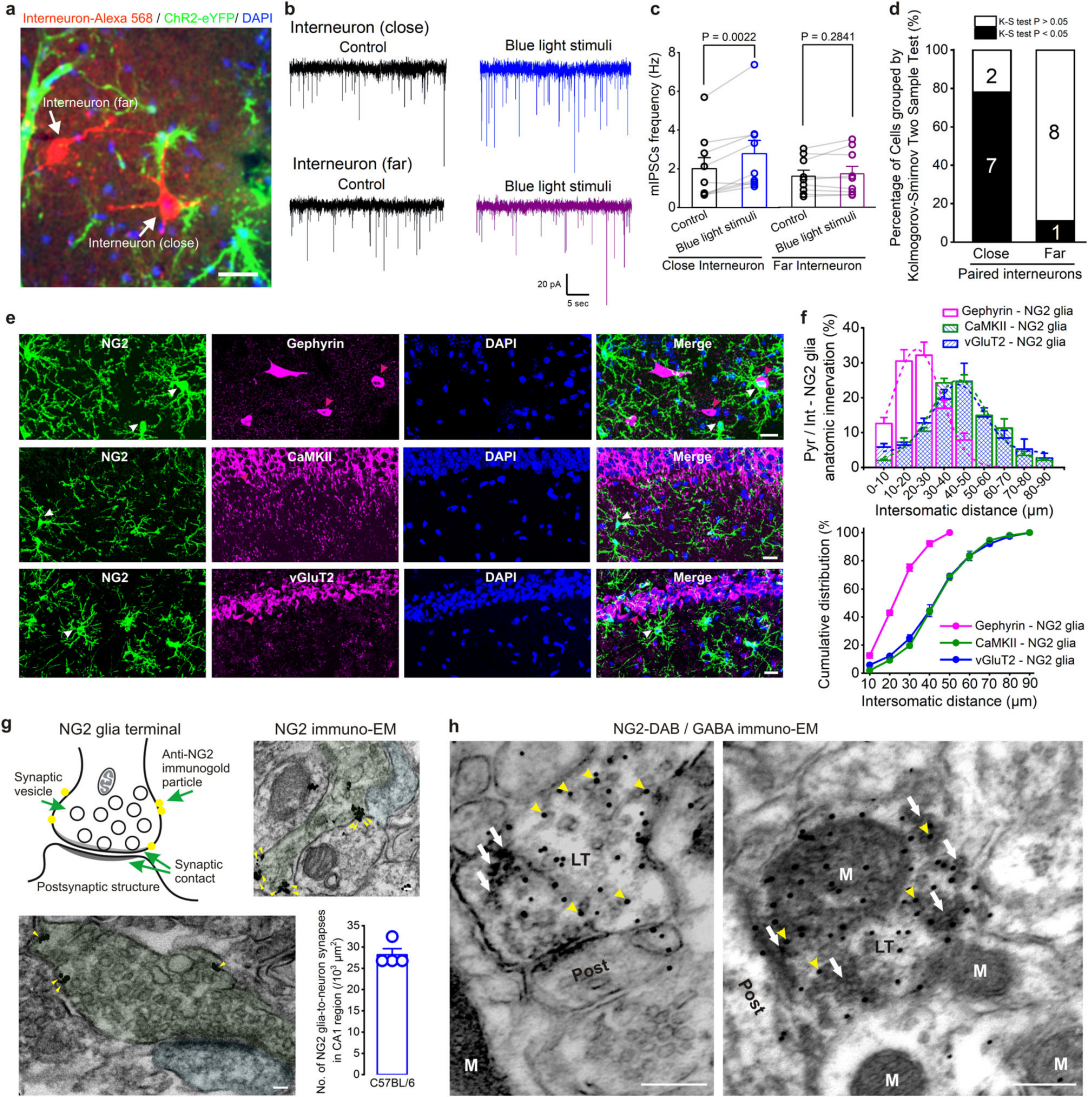
NG2 glia form pre- and postsynaptic structures with proximal hippocampal interneurons. **a** Representative image showing the morphology of dual-patched interneurons loaded with Alexa Fluor 568 in the patch pipettes in hippocampal CA1 region. One of the pair-recorded interneurons is typically chosen as the close interneuron, where the proximity to its closest NG2 glia soma is <30 µm, and the other is chosen as the far interneuron, where the proximity to its closest NG2 glia soma is farther than 30 µm. Scale bar, 20 μm. **b** Representative mIPSCs traces recorded from the close interneuron and far interneuron, showing an increase of frequency of mIPSCs typically occuring in the close interneuron after NG2 glia photostimulation (15 Hz, 90 s). **c** Summary bar graph shows a significant increase of frequency of mIPSCs onto the close interneurons but no effect on the far interneurons. *P* = 0.0022 and 0.2841 from 9 pairs for dual-patched close and far interneurons at postnatal 4–6 weeks, respectively, two-tailed paired *t* test. **d** Summary bar graph shows the percentage of cells displaying an increase of mIPSCs frequency from dual-recorded interneurons using Kolmogorov–Smirnov two-sample test analysis. The percentage of responsive cells is increased to 78% in the close interneurons after NG2 glia photoactivation. **e** Representative images showing immunostainings for gephyrin (upper panel, in magenta), CaMKII (middle panel, in magenta), and vGluT2 (lower panel, in magenta) with GFP-labeled NG2 cells (in green). The arrowheads in magenta and white indicate the anatomic proximity between the interneuron’s or pyramidal cell’s soma and its closest NG2 glia soma. Scale bars, 20 μm. **f** Histogram and cumulative distribution graph show the connection probabilities with respect to the inter somatic distance between gephyrin/CaMKII or vGluT2-positive neurons and NG2 glia. *n* = 142 pairs for gephyrin (+) interneurons–NG2 glia, *n* = 195 pairs, and *n* = 188 pairs for CaMKII (+) and vGluT2 (+) pyramidal neurons to NG2 glia from 3 mice at postnatal 6–12 weeks, respectively. **g** Cartoon illustrating a synaptic connection between an NG2 glial terminal and its postsynaptic dendrite from C57BL/6 wild-type mouse hippocampus. Representative images on the right panel and the panel below show NG2 antibody-labeled immunoelectron microscopy in C57BL/6 wild-type mouse hippocampus at postnatal 6–8 weeks. The presynaptic, postsynaptic structures, and the gold particles are indicated by green, blue and yellow, respectively. Scale bars, 50 nm. The bar graph shows the density of NG2-to-neuron synapses in CA1 region of the hippocampus (28.18 ± 1.42/10^3^ µm^2^), *n* = 4 mice. **h** Double labeling for GABA-immunogold within NG2-DAB positive processes/terminals, which are shown making a presynaptic contact with an underlying postsynaptic structure. The darkened “patchy/cloudy” DAB reaction products are seen mostly within the NG2-positive glial terminal (containing pleomorphic synaptic vesicles), using an antibody against NG2, as indicated by the white arrows. GABA-immunogold particles are labeled either within or adjacent to many synaptic vesicles (yellow arrowheads). LT indicates an NG2-labeled terminal. Post indicates postsynaptic structure. M indicates mitochondrion. Scale bars, 200 nm. All experiments are replicated from 5 mice. Data are presented as mean values ± SEM and error bar represents SEM.

It has been reported that cortical OPCs appear to position their cell bodies close to GABAergic neurons in particular and form OPC-neuron pairs, where GABA_A_ receptors are mainly clustered at inhibitory synapses on the soma and dendritic shafts of mature inhibitory neurons^38^. We first examined whether NG2 glia showed a similar topography with neurons in the hippocampal CA1 region using electrophysiological methods. We obtained dual whole-cell patch-clamp recordings by choosing a spatially far (>30 µm) or close (<30 µm) interneuron from one NG2 glial cell body, when approaching the photostimulation (Fig. 3a, b). Indeed, in a total of 9 paired recordings of interneurons, the successful rate of enhancement of mIPSCs frequency in close interneurons after NG2 glia photoactivation was largely increased to 78% (7 out of 9 close interneurons), yet not affecting mIPSCs frequency of far interneurons nor of pyramidal neurons (Fig. 3b–d and Supplementary Fig. 7, *n* = 9 pairs for each group). We further analyzed the spatial distribution between NG2 glia and adjacent interneurons or pyramidal neurons using immunohistochemistry. The gephyrin-labeled interneurons showed a maximum of 32.2 ± 3.7% proximity between 20 and 30 µm and a total of 75.2 ± 2.4% distribution, which is <30 µm to their closest NG2 glia somata (Fig. 3e, f and Supplementary Movie 1, *n* = 142 pairs)^38,39^. However, Ca^2+^/calmodulin dependent protein kinase II (CaMKII)-labeled or vesicular glutamate transporter 2 (vGluT2)-labeled excitatory pyramidal neurons in hippocampal CA1 region showed a total of 19.6 ± 1.6% or 24.9 ± 2.5% distribution, which is <30 µm to their closest NG2 glial somata (Fig. 3e, f, *n* = 195 and 188 pairs for CaMKII (+) and vGluT2 (+) pyramidal neurons to NG2 glia from 3 mice, respectively), suggesting that a highly spatial arrangement has an important role in NG2 glia-to-interneuron interactions.

To directly assess whether there are NG2 glia-to-neuron synapses at the ultrastructural level, we combined pre-embedded immunogold with EM to provide a precise synaptic structure to distinguish whether there is a direct synaptic junctional complex between the two cells at the nano-scale level. Using NG2 antibody immunogold labeling, we found that NG2 immunogold particles mainly distributed contiguously along the plasma membrane while there were still a few immunogold particles scattered in the cytoplasm of NG2 glia in the C57BL/6 wild-type mouse hippocampus (Supplementary Fig. 8b). To exclude non-specific immunogold labeling, the determination of a presynaptic NG2 glial processes/terminal, making a synaptic contact onto a postsynaptic neuronal structure was recognized with the following criteria: (1) dark and dense immunogold particles (diameter >10 nm) labeled with three or more along the plasma membrane; (2) presence of synaptic vesicles; (3) visually apparent synaptic cleft; and (4) identification of a postsynaptic density^40,41^. Surprisingly, we observed clear NG2 glia-to-neuron synaptic contact, where NG2 glial terminals contained neuronal-like presynaptic vesicles in the hippocampal CA1 region (Fig. 3g). As NG2 glia mainly exhibit a typical complex stellate morphology and have an evenly non-overlapping distribution in gray matters including hippocampus^42–44^, we estimated that one single NG2 glia could directly form about 146 presynaptic structures with neurons in the hippocampal CA1 region, in terms of the synaptic density of NG2 glia-to-neuron synapses (28.2 ± 1.4 per 10^3^ µm^2^, *n* = 4 mice, Fig. 3g) and the territory of NG2 glia (5191.3 ± 429.6 μm^2^, *n* = 6 analyzed NG2 cells, Supplementary Fig. 9). Moreover, 3,3’-diaminobenzidine (DAB) labelings for NG2 antibody localization combined with post-embedded GABA-immunogold methodology^45–47^, we further confirmed that presynaptic DAB-labeled NG2 processes/terminals contained GABA-immunogold particles either within or adjacent to many synaptic vesicles (Fig. 3h). Taken together, this anatomical micro-structural evidence from spatial proximity and immuno-EM, as well as the functional regulation of inhibitory synaptic activity, strongly supports a direct synaptic input from NG2 glia to hippocampal interneurons.

### GAD67 biosynthesis and VAMP-2-dependent vesicular exocytosis are required for GABA signal transduction in NG2 glia

**Fig. 4:**
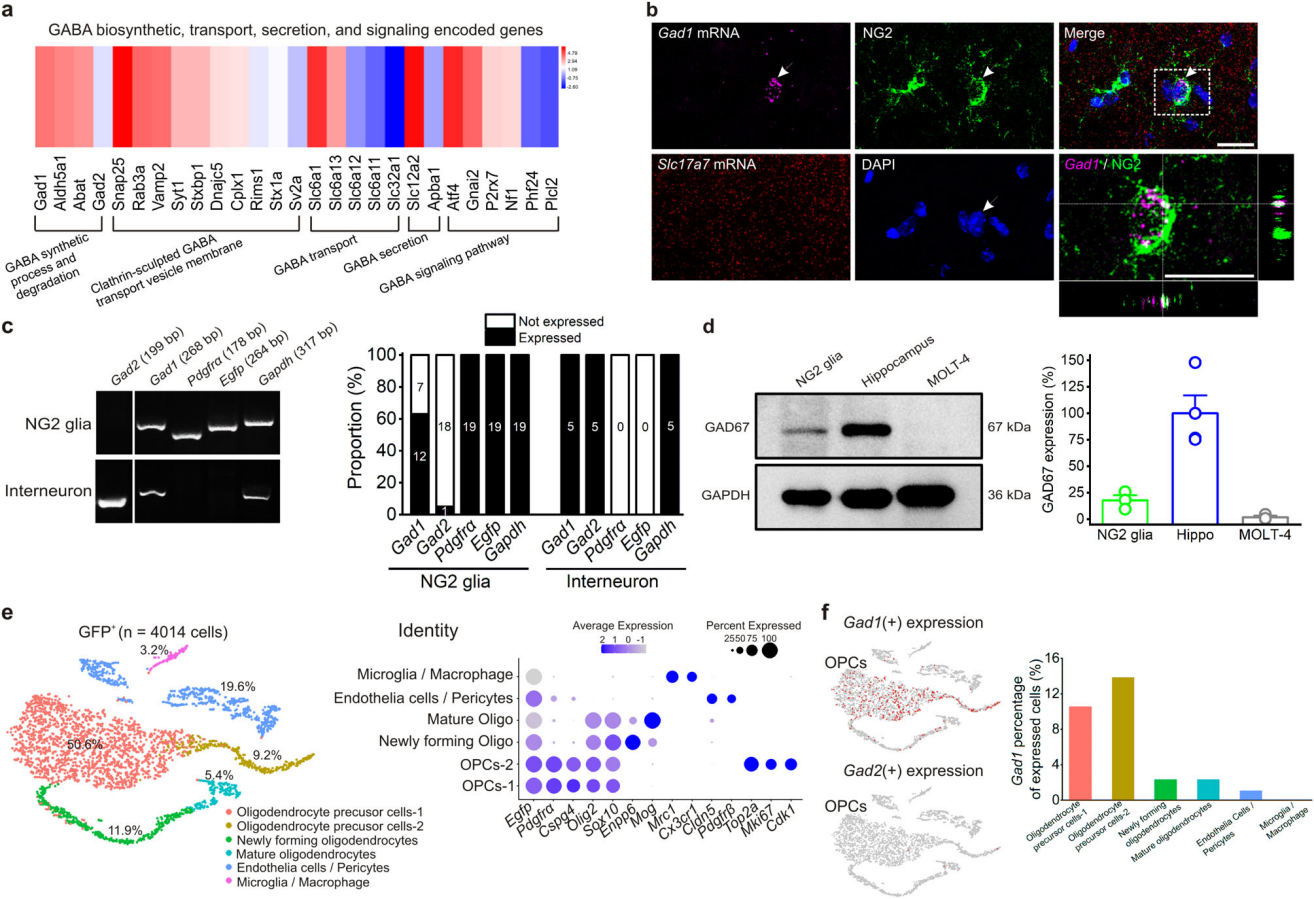
Transcriptomic analysis of *Gad1* expression in NG2 glia. **a** The gene expression levels with Log2 (FPKM) reveal a transcriptomic profile related to GABA synthetic processes and degradation, clathrin-sculpted GABA transport vesicle membrane, GABA transport, secretion, and GABA signaling pathway. Note that *Gad1* gene (GAD67) is relatively highly expressed in NG2 glia isolated from 3 Pdgfrα-creER^TM^;mGFP mice brains at postnatal 3–4 weeks. **b** Representative images of RNAscope with post-immunohistochemistry reveal a  colocalization of *Gad1* mRNA but not *Slc17a7* mRNA with an individual GFP-labeled NG2 glial cell. The colocalization image in the white dotted box is magnified as indicated with arrows. The image data are repeated from 3 mice at postnatal 4–6 weeks. Scale bars, 20 μm. **c** Representative images and bar graph summary showing single-cell RT-PCR analysis of *Gad1* and *Gad2* mRNA expression in NG2 glia with interneuron as its control from hippocampal CA1 region of Pdgfrα-creER^TM^;mGFP mice at postnatal 3–4 weeks. Note 12 out of 19 GFP (+) cells showed distinct *Gad1* but not *Gad2* expression in NG2 glia. **d** Representative images of western blot and bar graph summary showing GAD67 protein expression in the purified NG2 glia by FACS from 3 Pdgfrα-creER^TM^;mGFP mice brain at postnatal 3–4 weeks. GAD67 expression in NG2 glia is 17.81 ± 4.76% (*n* = 3) relative to hippocampal tissue (*n* = 4). MOLT-4 cells as a negative control (*n* = 3). **e** Single-cell RNA-sequencing analysis shows a t-distributed stochastic neighbor embedding (t-SNE) plot depicting the cell-type identity of total 4014 EGFP (+) cells by FACS from a Pdgfrα-creER^TM^;mGFP mouse hippocampus at postnatal 3 weeks. Six clusters identified on the basis of the expression of well-established marker genes are shown in the right graph. **f** t-SNE maps visualizing single-cell gene expression of *Gad1* and *Gad2* within six clusters. Note that *Gad1* but not *Gad2* mainly distributes in the OPC subclusters 1 and 2. Bar graph summarizes the percentage of *Gad1* expression within each cluster cells. Data are presented as mean values ± SEM and error bar represents SEM.

To determine the source of enhanced GABA levels in the synaptic cleft after NG2 glia photoactivation, we first performed bulk RNA-sequencing analysis to profile the genes related to GABA synthesis, trafficking, and metabolism in sorted NG2 glia by fluorescence-activated cell sorting (FACS) in Pdgfrα-creER^TM^;mGFP hippocampus (Supplementary Fig. 10a). In contrast to lower fragments per kilobase of exon per million fragments (FPKM) expression of a major glutamate transporter gene *Slc17a7* (encoding vesicular glutamate transporter 1, vGluT1)^48^, we found a relatively high FPKM expression of *Gad1* (encoding glutamic acid decarboxylase 67, GAD67) gene in NG2 glia, which mainly contributes to GABA biosynthesis (Fig. 4a and Supplementary Fig. 10b)^49,50^. To corroborate the above findings at a transcriptomic level, the RNAscope results combined with post-immunohistochemistry approach visualized an apparent expression of *Gad1* but not *Slc17a7* mRNA in individual GFP-labeled NG2 glial cells, as shown in Fig. 4b. To further determine the proportion of *Gad1* expression among NG2 glia and its possible distribution pattern, we performed two sets of examinations. First, we utilized single-cell RT-PCR analysis of *Gad1* and *Gad2* (encoding glutamic acid decarboxylase 65, GAD65) mRNA expression in NG2 glia from the hippocampal CA1 region of Pdgfrα-creER^TM^;mGFP mice at postnatal 4–6 weeks, with GFP(-) CA1 interneurons as the positive control. We found that 12 out of 19 GFP(+) NG2 glia showed distinct *Gad1* but not *Gad2* expression in situ (Fig. 4c, *n* = 19 and 5 for NG2 glia and interneurons, respectively). Moreover, western blot analysis revealed GAD67 protein expression in sorted NG2 glia by FACS in Pdgfrα-creER^TM^;mGFP hippocampus (Fig. 4d, *n* = 3 mice at postnatal 3–4 weeks). Second, we utilized FACS method to isolate GFP(+) NG2 glial cells from a Pdgfrα-CreER^TM^;mGFP mouse hippocampus and examined single-cell transcriptomic profiles. From the total 4014 GFP(+) cells, the t-SNE plot data showed 6 clusters by cell identity, which contain a 77.2% percentage of oligodendrocyte lineage cells (Fig. 4e). The t-SNE maps visualized the scattered expression of single-cell gene *Gad1* but not *Gad2* within OPC subclusters 1 and 2, which were mainly divided by the cell division cycle genes *Top2a*, *Mki67,* and *Cdk1* (Fig. 4f).

**Fig. 5:**
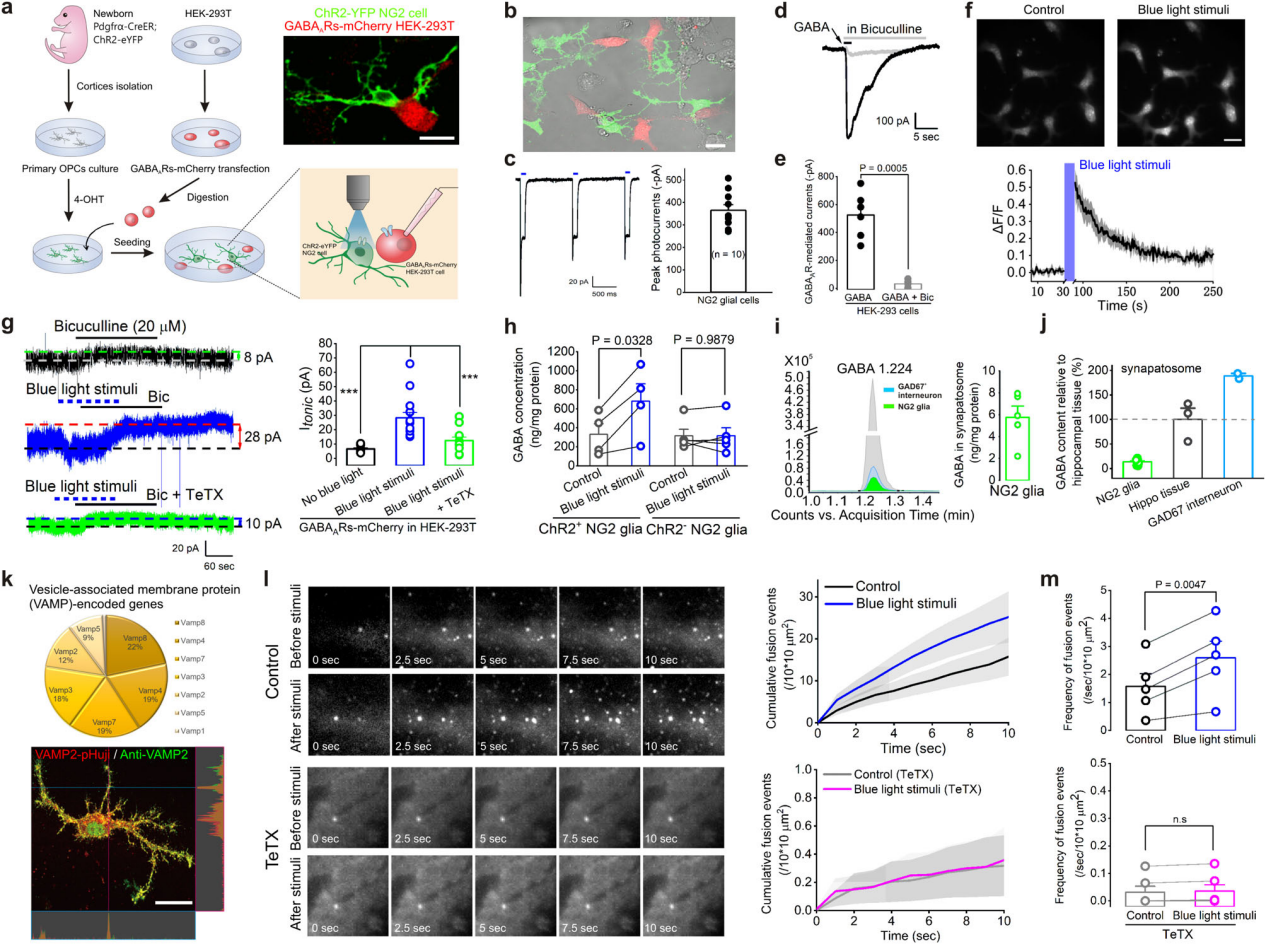
NG2 glia relay GABA signals through VAMP-2-dependent vesicular exocytosis. **a** Cartoon images illustrating the sniffer-patch method in co-cultured NG2 cells and HEK-293T cells. Scale bar, 20 μm. **b** Representative image showing primary cultured NG2 cells (in green) from Pdgfrα-creER^TM^; ChR2-eYFP newborn mice brain with GABA_A_Rs-mCherry transfected HEK-293T cell (in red). Scale bar, 20 μm. **c** The representative trace shows an example of ChR2-evoked photocurrents by 100 ms blue light stimulation in a cultured NG2 cell. Bar graph shows −365.3 ± 25.1 pA peak photocurrent induced by blue light stimulation in cultured NG2 cells, *n* = 10 cells. **d**, **e** Representative traces (**d**) and summarized bar graph (**e**) show that 25 μM GABA application induces a distinct GABA_A_R-mediated current that is significantly abolished by GABA_A_R antagonist bicuculline in transfected HEK-293T cells. GABA_A_R-mediated currents: control: −526.2 ± 67.2 pA, *n* = 6 cells; in the presence of bicuculline: −32.9 ± 10.7 pA, *n* = 6 cells, two-tailed paired *t* test, *P* = 0.0005. **f** Representative images show robust [Ca^2+^]_i_ elevations in cultured NG2 cells loaded with the calcium indicator Rhod2-AM (5 µM) after 15 Hz 60 s blue light stimulation. Bar graph summary below shows the time course of blue light stimulation-induced [Ca^2+^]_i_ increase in cultured NG2 cells. *n* = 53 cells from 3 mice. Data are normalized by the mean fluorescence intensity obtained during the control period (0–30 s before the stimulation triggers) for each cell. **g** Representative traces and summarized bar graph show a significant increase of GABA_A_R-mediated tonic current in sniffer-patched transfected HEK-293T cell in the presence of blue light stimulation of cultured NG2 cells (middle panel) compared with its basal tonic inhibition in the absence of blue light stimulation (upper panel) obtained from a Pdgfrα-creER^TM^;ChR2 mouse. This increased tonic GABA current is abolished by the cotransfection with exocytosis blocker TeTX in the presence of NG2 cells photoactivation (lower panel). Basal tonic GABA currents in transfected HEK cells without blue light stimuli: 6.57 ± 0.82 pA, *n* = 10 cells; tonic GABA currents in the presence of blue light stimuli: 28.27 ± 3.61 pA, *n* = 15 cells; tonic GABA currents in the presence of TeTX and blue light stimuli: 12.38 ± 2.33 pA, *n* = 12 cells. ANOVA Tukey–Kramer Multiple Comparisons, *** indicates *P* < 0.001. **h** Bar graph summary showing increased GABA concentrations in purified ChR2-expressing NG2 cells after blue light stimulation compared with its control with high-performance liquid chromatography (HPLC) analysis. *n* = 4 and 5 independent experiments for ChR2^+^-NG2 cells and ChR2^-^-NG2 cells, respectively. *P* values as indicated, two-tailed paired *t* test. **i** GABA levels in synaptosomes isolated from purified NG2 cells and GAD67-GFP interneurons with HPLC. Bar graph showing an average GABA concentration of 5.76 ± 1.04 ng/mg synaptosomes in NG2 glia (right panel). *n* = 5 tested samples. **j** The bar graph summary shows GABA contents in synaptosomes in both purified NG2 cells and GAD67+ interneurons compared with hippocampal tissue. *n* = 11 tested samples for purified primary cultured NG2 cells, *n* = 2 and 3 tested samples for GAD67 interneurons and hippocampi from 4 GAD67-GFP and 3 C57BL/6 mice, respectively. **k** The pie graph shows a component percentage of vesicle-associated membrane protein (VAMP)-encoded genes through RNA-sequencing data analysis of isolated NG2 glia in adult brain by FACS at postnatal 3–4 weeks. The representative image below shows a precise colocalization of transfected VAMP2-pHuji plasmid (in red) with anti-VAMP2 antibody (in green) in primary cultured NG2 cells. Scale bar, 20 μm. **l** Representative TIRFM images show the total VAMP2-pHuji laden vesicular fusion events at 0, 2.5, 5, 7.5, and 10 s with the image size of 10 × 10 μm^2^ before (upper panel) and after (lower panel) blue light stimulation (10 Hz, 60 s) in the absence (control panels) or the presence of TeTX (TeTX panels), respectively. The average cumulative graphs of fusion events during a 10 s time window before and after blue light stimulation in the absence (control) or the presence of TeTX are shown on the right. **m** Summarized bar graphs show a significant enhancement of exocytosis of VAMP-2 laden vesicles in cultured NG2 cells after blue light stimulation. In TeTX, this increased exocytosis is completely abolished by the exocytosis blocker TeTX. Exocytosis events are analyzed from 5 and 6 NG2 cells for control and TeTX group, respectively. *P* value as indicated, two-tailed paired *t* test, n.s. indicates not significant. Data are presented as mean values ± SEM and error bar represents SEM.

As NG2 glia express GABA synthetase GAD67 and contain significant amounts of synaptic vesicles based on the aforementioned transcriptomic and immuno-EM results (Figs. 3g, h, 4a–f and Supplementary Fig. 8), it was highly possible that NG2 glia could functionally release GABA through a vesicular transduction machinery. Thus, to directly test this idea, we introduced a sniffer-patch technique with co-cultured purified ChR2-expressing NG2 cells and GABA_A_Rs-transfected HEK-293T cells in vitro to exclude potential confounds from either neurons or astroglia which could cause GABA release in brain tissue^51,52^. The rationale is that if activation of NG2 cells drives GABA transmitter release by optogenetic stimulation, one could detect GABA-induced tonic inhibition in an adjacent GABA_A_Rs-transfected HEK-293T cell. As described in the schematic experimental procedure shown in Fig. 5a, we successfully recorded both reliable optogenetically-induced photocurrents in ChR2-eYFP-expressing cultured NG2 cells (Fig. 5b, c, *n* = 10 cells, and Supplementary Fig. 11) and GABA (25 µM)-induced inward currents in GABA_A_Rs-mCherry transfected HEK-293T cells (Fig. 5d, e, *n* = 6 cells). In addition, the purified NG2 cells exhibited a robust [Ca^2+^]_i_ elevation after 15 Hz 60 sec blue light stimulation, and this [Ca^2+^]_i_ increase was not abolished in the presence of TTX when the cells were loaded with the calcium indicator Rhod2-AM (5 µM) (Fig. 5f and Supplementary Fig. 12, *n* = 53 cells from 3 mice). With the aid of sniffer-patch method^52,53^, we indeed found an ~4.3-fold increase of tonic GABA inhibition in GABA_A_Rs-mCherry transfected HEK-293T cells, which occurred only after photoactivation of NG2 cells (Fig. 5g, *n* = 10 and 15 cells for basal control and blue light stimuli, respectively, *P* < 0.001). In addition, this enhancement of tonic current was significantly diminished in the presence of an exocytosis blocker, tetanus toxin (TeTX), indicating a vesicular GABA release signal transduction mechanism (Fig. 5g, *n* = 12 cells transfected with TeTX).

Next, we examined whether the release of GABA was required for synaptic vesicular trafficking. First, we tested the change of GABA levels in purified ChR2-expressing NG2 cells before and after blue light stimulation. With the aid of high-performance liquid chromatography (HPLC) analysis, an increased GABA level in purified ChR2-expressing NG2 cells occurred after blue light stimulation compared with its control in the absence of photostimulation (331.67 ± 111.37 ng/mg for control vs. 681.48 ± 181.27 ng/mg, *n* = 4 independent experiments, *P* = 0.0328, two-tailed paired *t* test, Fig. 5h). Second, we directly isolated the synaptosomes in purified NG2 cells and measured the GABA content of an average of 5.76 ± 1.04 ng/mg synaptosomes (*n* = 5 tested samples, Fig. 5i) with GAD67-GFP interneurons and hippocampal tissue as the controls (Fig. 5j, *n* = 2 and 3 tested samples for GAD67 interneurons and hippocampi from 4 GAD67-GFP and 3 C57BL/6 mice, respectively). Third, we evaluated the vesicular trafficking-related genes for neurotransmitters from our transcriptomic profiling. To our surprise, NG2 glia contains a soluble *N*-ethylmaleimide-sensitive fusion protein attachment protein receptor (SNARE) complex, including the members VAMP (vesicle-associated membrane protein) and SNAP-25 (synaptosomal-associated protein 25), which are thought to be crucial for neuronal synapse formation, development, and trafficking (Fig. 5k and Supplementary Fig. 10c)^53–55^. Since VAMP-2-containing vesicles mainly express in neurons and astrocytes and play an essential role in neurotransmitter storage such as GABA, glutamate, or ATP^54–57^, whereas *VAMP2* mRNA expression constitutes a large percentage of vesicular trafficking protein encoded genes in NG2 glia (Fig. 5k and Supplementary Fig. 10c), we transfected fluorescent synaptobrevin 2 (VAMP-2) derivative, synapto-pHuji plasmids into purified NG2 cells to visualize the VAMP-2-labeled vesicular fusion events on membrane trafficking with total internal reflection fluorescence microscopy (TIRFM)^56–59^. As shown in Fig. 5k, the transfected VAMP-2-containing vesicles in red fluorescence were largely colocalized with anti-VAMP-2 antibody as shown by green puncta. After photoactivation of ChR2-expressing NG2 cells by blue light stimuli, the VAMP-2 laden vesicles showed a substantial increase of mobility and fusion events, indicating an enhanced VAMP-2 vesicular exocytosis occurred after NG2 cell activation (the frequency of VAMP-2 vesicle fusion events is: 1.58 ± 0.46/sec/100 µm^2^, *n* = 1892 vesicles before, vs. 2.59 ± 0.60/sec/100 µm^2^, *n* = 3121 vesicles after photostimulation from 5 NG2 cells, two-tailed paired *t* test, *P* = 0.0047, Fig. 5l, m). In contrast, NG2 cells transfected with exocytosis blocker TeTX not only showed a significant reduction of VAMP-2-laden vesicles, but also the increased number of fusion events was completely abolished compared with the control group (the frequency of VAMP-2 vesicle fusion events in TeTX is: 0.03 ± 0.02/sec/100 µm^2^, *n* = 92 vesicles before, vs. 0.04 ± 0.02/sec/100 µm^2^, *n* = 103 vesicles after photostimulation from 6 NG2 cells, two-tailed Wilcoxon-matched pairs test, *P* = 0.125, Fig. 5l, m, and Supplementary Movie 2). Taken together, these results provide undisputed evidence that activation of NG2 glia mainly recruits a GAD67 synthase and VAMP-2 laden vesicular trafficking machinery to effect a neurotransmitter GABA release process.

### Increased calcium signals in NG2 glia and enhanced anxiety-like behavior in CSDS model mice

**Fig. 6:**
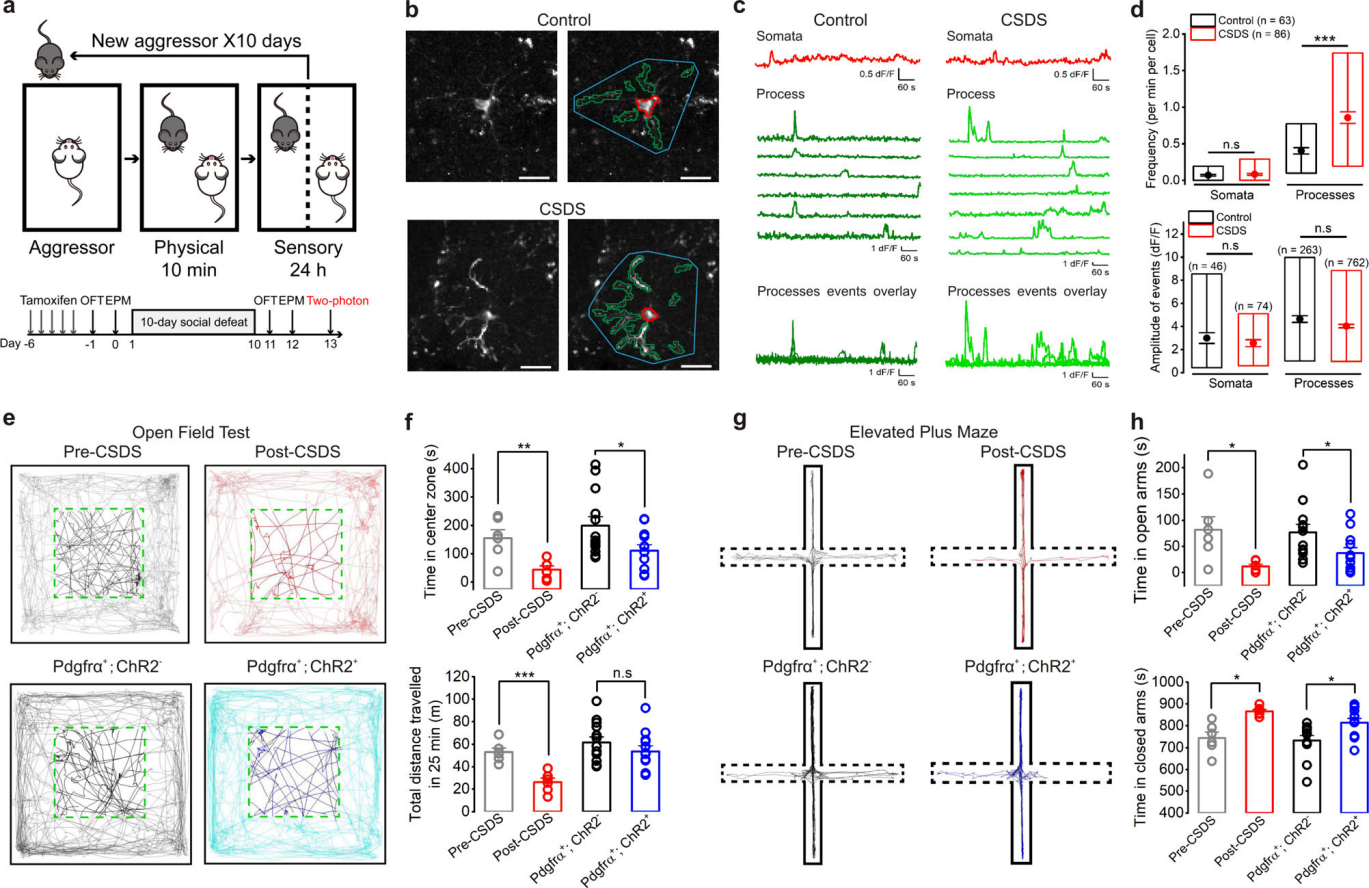
Increased calcium signals in NG2 glia and enhanced anxiety-like behavior in chronic social defeat stress (CSDS) mice. **a** Schematic illustrating the experimental approach for calcium imaging and behavioral tests. After 10 consecutive days of CSDS, experimental animals were housed singly to perform the following experiments. **b**, **c** Representative images (**b**) and traces (**c**) of Ca^2+^ fluctuations measured in hippocampal NG2 glia from a control mouse and CSDS mouse at postnatal 8–12 weeks. Two predominant types of Ca^2+^ events are defined: somatic fluctuations (red), process waves (olive for control and green for CSDS). Approximate territory boundaries are outlined in blue, but these were not used for data analyses and are shown only for illustrative purposes. Scale bars, 20 µm. **d** Average data for Ca^2+^ fluctuation properties in control and CSDS mice (from 3 mice for each group). The frequency of Ca^2+^ fluctuations per min per cell is shown in upper panel: somata of control, 0.07 ± 0.01 vs. somata of CSDS, 0.08 ± 0.01, *P* = 0.9845, two-tailed Mann–Whitney test; processes of control, 0.40 ± 0.04 vs. processes of CSDS, 0.86 ± 0.08; *P* < 0.0001, two-tailed Mann–Whitney test, *** indicates *P* < 0.001, n.s. indicates not significant, *n* = 63 and 86 cells for control and CSDS, respectively. The amplitude of Ca^2+^ fluctuations is shown in lower panel: somata of control, 2.99 ± 0.47 vs. somata of CSDS, 2.55 ± 0.30, *P* = 0.9162, two-tailed Mann–Whitney test; processes of control, 4.63 ± 0.29 vs. processes of CSDS, 4.03 ± 0.15, *P* = 0.0799, two-tailed Mann–Whitney test, n.s. indicates not significant, the *n* refers to the number of Ca^2+^ fluctuations for control and CSDS. Bounds of box: 10th and 90th percentile and whisker: SEM. **e** Representative open-field activity tracks show the effects of optical stimulation in CSDS mice and its control group; Pdgfrα-creER^+^;ChR2^+^ mice group and its Pdgfrα-creER^+^;ChR2^−^ control group in a period of 25 min including blue light stimulations (20 Hz, 20 msec, 10 s ON/OFF). **f** The summary bar graphs show the time in center zone (upper panel) and the distance (lower panel) traveled by the mice over 25 min in an open-field chamber for the two sets of experimental groups. Time in center zone: 155.59 ± 29.80 sec in Pre-CSDS vs. 43.96 ± 13.29 sec in Post-CSDS, *n* = 6 mice at postnatal 8–12 weeks, two-tailed paired *t* test, *P* = 0.0083; 208.81 ± 32.54 sec in Pdgfrα-creER^+^;ChR2^−^ vs. 111.22 ± 21.06 sec, *n* = 12 mice at postnatal 8–12 weeks for each group, two-tailed Mann–Whitney test, *P* = 0.0332. Total distance traveled in 25 min: 53.06 ± 3.69 m in Pre-CSDS vs. 26.21 ± 3.67 m in Post-CSDS, *n* = 6 mice at postnatal 8–12 weeks, two-tailed paired *t* test, *P* = 0.0003; 61.13 ± 5.54 m in Pdgfrα-creER^+^;ChR2^−^ vs. 53.68 ± 4.99 m, *n* = 12 mice at postnatal 8–12 weeks for each group, two-tailed unpaired *t* test, *P* = 0.3282. **g** Representative elevated plus maze activity tracks show the effects of optical stimulation between CSDS mice and its control group; Pdgfrα-creER^+^;ChR2^+^ mice group and its Pdgfrα-creER^+^;ChR2^−^ control group in a period of 15 min including blue light stimulations (20 Hz, 20 msec, 10 s ON/OFF). **h** The summary bar graphs show the time spent in the open arms (upper panel) and the time spent in the closed arms (lower panel) traveled by the mice over 15 min in an elevated plus maze for the two sets of experimental groups. Time in open arms: 81.64 ± 25.06 sec in Pre-CSDS vs. 11.83 ± 4.13 sec in Post-CSDS, *n* = 6 mice at postnatal 8–12 weeks, two-tailed paired *t* test, *P* = 0.0458; 76.90 ± 15.56 sec in Pdgfrα-creER^+^;ChR2^−^ vs. 37.18 ± 10.57 sec, *n* = 12 mice at postnatal 8–12 weeks for each group, two-tailed unpaired *t* test, *P* = 0.0463. Time in closed arms: 743.98 ± 27.99 sec in Pre-CSDS vs. 866.59 ± 8.70 sec in Post-CSDS, *n* = 6 mice at postnatal 8–12 weeks, two-tailed paired *t* test, *P* = 0.0112; 732.59 ± 22.73 sec in Pdgfrα-creER^+^;ChR2^−^ vs. 814.55 ± 19.62 sec, *n* = 12 mice at postnatal 8–12 weeks for each group, two-tailed Mann–Whitney test, *P* = 0.0173. * indicates *P* < 0.05, ** indicates *P* < 0.01, *** indicates *P* < 0.001, n.s. indicates not significant. Data are presented as mean values ± SEM and the error bar represents SEM.

**Fig. 7:**
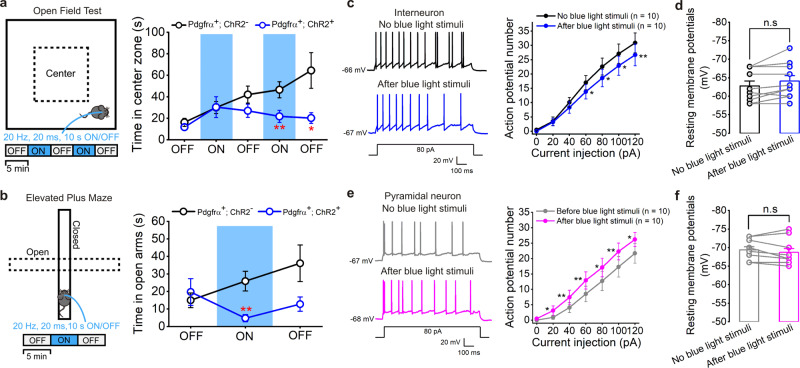
NG2 glia photoactivation triggers an anxiety-like behavior through an E-I balance perturbation in adult mice. **a** Cartoon illustrating the procedure for intermittent photoactivation of ChR2-expressing NG2 glia in the adult hippocampus at postnatal 8–12 weeks in an open field test. Pdgfrα-creER^+^; ChR2^−^ mice as its negative control. The right panel shows an average time in the center zone traveled by the mice over 25 min, including blue light stimulations (20 Hz, 20 msec, 10 s ON/OFF) in an open field chamber, divided into 5-min epochs for the two experimental groups. Time in center zone: 17.23 ± 3.98 sec vs. 11.72 ± 2.72 sec in 0–5 min OFF, *P* = 0.2649; 31.92 ± 5.73 sec vs. 30.44 ± 9.66 sec in 5–10 min ON, *P* = 0.8961; 43.82 ± 8.33 sec vs. 27.03 ± 6.12 sec in 10–15 min OFF, *P* = 0.1187; 48.99 ± 7.73 sec vs. 21.80 ± 5.62 sec in 15–20 min ON, *P* = 0.0094; 66.86 ± 17.83 sec vs. 20.24 ± 4.95 sec in 20–25 min OFF, *P* = 0.0242. *n* = 12 for Pdgfrα-creER^+^;ChR2^−^ and Pdgfrα-creER^+^;ChR2^+^ mice at postnatal 8–12 weeks in each group. Two-tailed unpaired *t* test (0–5, 5–10, 10–15, 15–20 min), two-tailed Mann–Whitney test (20–25 min). * indicates *P* < 0.05, ** indicates *P* < 0.01. **b** Cartoon illustrating the procedure for intermittent photoactivation of ChR2-expressing NG2 glia in the adult hippocampus at postnatal 8–12 weeks in an elevated plus maze test. Pdgfrα-creER^+^; ChR2^−^ mice as its negative control. The right panel shows an average time in the open arms traveled by the mice in a period of 15 min, including blue light stimulation (20 Hz, 20 msec, 10 s ON/OFF) in an elevated plus maze, divided into 5-min epochs for the two experimental groups. Time in open arms: 14.95 ± 4.21 sec vs. 19.67 ± 7.62 sec in 0–5 min OFF, *P* = 0.9309; 25.88 ± 5.62 sec vs. 4.74 ± 2.04 sec in 5–10 min ON, *P* = 0.0013; 36.07 ± 10.46 sec vs. 12.77 ± 4.11 sec in 10–15 min OFF, *P* = 0.0776. *n* = 12 for Pdgfrα-creER^+^;ChR2^−^ and Pdgfrα-creER^+^;ChR2^+^ mice at postnatal 8–12 weeks in each group. Two-tailed Mann–Whitney test, ** indicates *P* < 0.01. **c** Representative traces of action potentials (APs) generated in an interneuron in hippocampal CA1 region showing reduced membrane excitability after NG2 glia are activated by repetitive photostimulation (15 Hz, 4 min). The summary graph in the right panel shows an average AP firing rate at different current injections (current injections are given in a total of 25 depolarizing steps with 5 pA increments and 1000 ms in duration) in interneurons before and after NG2 glia photostimulation. AP firing numbers at 60, 80, 100, and 120 pA current injection are 17 ± 2.5, 22.6 ± 3.0, 27.1 ± 3.4, 30.9 ± 3.5 vs. 13.8 ± 2.5, 18.6 ± 3.1, 22.9 ± 3.3, 26.7 ± 3.8, *P* = 0.0147, 0.015, 0.0102, 0.0044, respectively. * indicates *P* < 0.05, ** indicates *P* < 0.01, two-tailed paired *t* test, *n* = 10 cells. **d** The bar graph shows no change of resting membrane potentials of interneurons before and after NG2 glia photostimulation. n.s. indicates not significant, two-tailed paired *t* test, *n* = 10 cells. **e** The same as in **c** but for hippocampal CA1 pyramidal neurons. Note pyramidal neurons show increased membrane excitability after NG2 glia photoactivation. AP firing numbers before and after NG2 glia photostimulation at 20, 40, 60, 80, 100, and 120 pA current injection are 0.9 ± 0.6, 4.1 ± 1.6, 8.5 ± 2.5, 12.7 ± 3.1, 17.3 ± 3.3, 21.7 ± 3.2 vs. 3.1 ± 1.5, 7.4 ± 2.3, 12.9 ± 2.8, 17.1 ± 3.0, 22.3 ± 2.7, 26.2 ± 2.3. *P* = 0.0313, 0.0046, 0.0031, 0.0102, 0.002, 0.0195, respectively. * indicates *P* < 0.05, ** indicates *P* < 0.01, two-tailed paired *t* test and Wilcoxon-matched pairs test, *n* = 10 cells. **f** The bar graph shows no change of resting membrane potentials of pyramidal neurons before and after NG2 glia photostimulation. n.s. indicates not significant, two-tailed paired *t* test, *n* = 10 cells. Data are presented as mean values ± SEM and the error bar represents SEM.

Social stressors are the main source of stress in humans and contribute to the development and expression of diverse pathologies including prominently anxiety disorder, which is prevalent worldwide and there are no satisfactory therapeutic options available to date^60,61^. Although chronic social stress has been attributed to the hippocampus, prefrontal cortex, amygdala activity^62^, and peripheral immune system response^63^, the pathological mechanism of anxiety disorders are still poorly understood. In a commonly used paradigm termed CSDS mouse model (Fig. 6a)^64,65^, we first evaluated intracellular Ca^2+^ signaling of NG2 glial cells using NG2-creER^TM^;GCaMP6s transgenic mice. In normal control mice, hippocampal NG2 glia displayed spontaneous yet slow [Ca^2+^]_i_ fluctuations (Fig. 6b–d, *n* = 63 and 86 cells for control and CSDS, respectively). However, a distinct increment of [Ca^2+^]_i_ fluctuations occurred mainly in NG2 glial processes after mice were exposed to social defeat stress (Fig. 6b–d and Supplementary Movie 3, *n* = 63 and 86 cells for control and CSDS, respectively), indicating an intrinsic activation of NG2 glia in CSDS. As social defeat has previously been shown to enhance anxiety-like behavior in mammalians^66–68^, we used the open field test (OFT) and elevated plus maze test (EPM) to examine the behavioral changes before and after the mice developed CSDS. Indeed, we found that mice spent significantly less time in the center zone of the open field arena (Fig. 6e, f, *n* = 6 mice at postnatal 8–12 weeks for Pre-CSDS and Post-CSDS group; *n* = 12 mice at postnatal 8–12 weeks for Pdgfrα^+^;ChR2^+^ and Pdgfrα^+^;ChR2^−^ each group), and this heightened anxiety in CSDS was further supported by their spending less time exploring the open arms in EPM test (Fig. 6g, h, *n* = 6 mice at postnatal 8–12 weeks for Pre-CSDS and Post-CSDS group; *n* = 12 mice at postnatal 8–12 weeks for Pdgfrα^+^;ChR2^+^ and Pdgfrα^+^;ChR2^−^ each group). To our surprise, this anxiety-like behavior in CSDS mice was closely replicated when simple photoactivation of hippocampal NG2 glia was applied in vivo without affecting the mice locomotor activity or social avoidance behavior (Figs. 6e–h, 7a, b, Supplementary Figs. 13, 14 and Supplementary Movies 4, 5), suggesting NG2 glia activation directly triggers anxiety-like behavior.

Excitatory-inhibitory (E-I) balance perturbation or GABAergic system dysregulation in the hippocampus can cause diverse severe neurological diseases including stress and emotional disorders^69–71^. To gain more insights as to how NG2 glia photoactivation regulates hippocampal network, we further assessed the membrane excitability of hippocampal interneurons in acute brain slices as our aforementioned electrophysiological results indicated an enhancement of mIPSCs frequency and tonic inhibition in adjacent interneurons of CA1 region (Fig. 2). As shown in Fig. 7c (*n* = 10 cells), the membrane excitability of interneurons was largely inhibited as AP firing rate was significantly reduced after NG2 glia were activated by continuous, 4-min photostimulation, which led to the accumulation of GABA at the synapse. In contrast, the AP firing rate of pyramidal neurons showed a significant increase after NG2 glia photostimulation without changing the resting membrane potentials of both groups of neurons (Fig. 7d–f, *n* = 10 cells for each group), indicating the E-I balance in the hippocampal circuit was disturbed. Taken together, calcium imaging in CSDS, electrophysiological evidence, and behavioral tests, show that experimental activation of NG2 glia, characterized by increased Ca^2+^ signals, induces anxiety-like behavior in a CSDS mouse model, potentially caused by an E-I balance perturbation. Whether this NG2 glial pathway is necessarily required in mediating CSDS remains to be further determined.

## Discussion

In summary, our work provides evidence that selective optogenetic activation of NG2 glia induces GABA neurotransmitter release and thereby regulates postsynaptic inhibitory activity specifically onto adjacent interneurons in adult mouse hippocampus. This ultimately triggers anxiety-like behavior through an E-I balance perturbation, which might play a role in CSDS. Different from astrocyte processes, which have been shown to wrap around axon terminals, forming the tripartite synapse^13,15,17^, NG2 glia are able to play a presynaptic neuron-like role by regulating/modulating inhibitory synaptic activity, as shown here using electrophysiology, immunoelectron microscopy, and TIRF microscopy, demonstrating vesicular compartments and increased VAMP-2-containing vesicular exocytosis after their activation (Figs. 2, 3 and 5). The classical concept of synapses formed by pre- and postsynaptic neurons was recently challenged by showing that glutamate transmission presents in non-neuronal cytoneme synapses of drosophila and synaptic integration of glioma and neurons^41,72^. Our findings herein provide a new perspective by demonstrating that NG2 glia-to-neuron synapses exist in the mammalian brain and shed new insights into our understanding of how glial cells integrate into neural circuits and the mutual crosstalk at the synaptic level in the CNS.

It is worth mentioning that there are two recent studies highlighting the fact that NG2 glia could regulate or modulate neuronal synaptic activity based on the following evidence, (1) NG2 cleavage by the α-secretase ADAM10 in OPCs impairs *N*-methyl-d-aspartate (NMDA) receptor-dependent long-term potentiation (LTP) in pyramidal neurons of somatosensory cortex and α-amino-3-hydroxy-5-methyl-4-isoxazole propionic acid (AMPA) receptor-mediated currents in these neurons are altered in NG2-knockout mice^73^; (2) The regionally selective depletion of NG2 glia with inducible expression of DT derivatives compromises AMPA receptor membrane trafficking, spontaneous miniature excitatory postsynaptic currents, and astrocytic glutamate uptake^74^. However, another study argued that such synaptic effects on postsynaptic transmission in neurons after NG2 glia ablation were not observed^75^. This debate could arise due to consequential changes of ion channels and/or membrane receptors in NG2 glia after genetic ablation of these glial cells^11,76^. The algal protein ChR2 is a rapidly light-gated cation channel, which is highly permeable to monovalent cations such as Na^+^, K^+^ and H^+^^24^. Through precisely controlling the membrane potential and firing activity of specific neurons by driving extracellular Na^+^ influx of the cell, ChR2 has been widely used to study neural circuits and brain functions^25,26^. Although NG2 glia does not generate APs, this unique type of cell demonstrates [Ca^2+^]_i_ elevation through the cooperation of sodium channels and sodium-calcium exchangers (Na^+^-Ca^2+^ exchangers, NCXs) during GABA-induced cell depolarization^77^. It has been reported that through the coordination of sodium influx via ChR2 channels and reversal of NCXs, astrocytes clearly showed the [Ca^2+^]_i_ elevation and that increased [Ca^2+^]_i_ could lead to Ca^2+^-dependent glial transmitters release including ATP, glutamate, and d-serine in astrocytes^29,30^. Indeed, in our study, we found that acute NG2 glia from hippocampal slices exhibited cell membrane depolarizations upon optogenetic stimulation (Supplementary Fig. 2). Further Ca^2+^ imaging in purified NG2 glial cells revealed a potential signaling pathway through the coordination of sodium channels and reversal of NCXs upon sodium influx via ChR2 channels (Fig. 5f and Supplementary Fig. 11), which suggests that this specific characteristic of NG2 glia could be physiologically utilized. In addition, in a social defeat stress mouse model, our study demonstrated that [Ca^2+^]_i_ elevation in NG2 glia could also occur in a potentially pathological circumstance (Fig. 6a–d). In fact, our previous study demonstrated that NG2 glia exhibited a depolarized resting membrane potential in pathologic conditions such as brain ischemia^11^. Therefore, owing to its high permeability to Na^+^, optogenetic stimulation of ChR2 channel may be an ideal way to mimic the cytosolic Na^+^ dynamics in NG2 glia that occurs during physiological and/or pathological processes.

Over the past 10 years, an increasing number of studies have revealed that GABAergic projection neurons are highly diverse in terms of molecular marker expression, activity pattern, synaptic targeting, and precise temporal recruitment^78^. As GABAergic projection neurons connect diverse brain areas unidirectionally or bidirectionally, they participate in the modulation of a whole series of behavioral and cognitive functions mostly seen in a distant or long-range endeavor. However, the mechanism of fine-tuning activity of these GABAergic neurons in a local and/or at a micro-range distance has not been well defined. It has been reported that NG2 glia/OPCs receive temporal and spatial innervation from cortical PV positive interneurons for oligodendrogenesis and maturation/differentiation during early brain development^39^. A local microcircuit architecture with interneuron-NG2 cell intersomatic distances never exceeded 70 μm, where fast-spiking interneurons (FSIs) contact preferentially NG2 cell somata and proximal branches^38,39^. In accord with this, we also observed a similar spatial cell–cell contact relationship in the adult hippocampus where adjacent interneurons are prone to respond to and process synaptic signals quickly from NG2 glia. An increased probability of successful synaptic innervation from dual-patch recordings occurred when NG2 glia-interneuron somatic distances were anatomically <30 μm in the hippocampal CA1 region (Fig. 3 and Supplementary Fig. 7), hence to accumulate a critical amount of vesicular GABA neurotransmitter released into the synaptic cleft to activate synaptic and extrasynaptic GABA_A_Rs after NG2 glia activation^79^. In the current study, we can not specify an exact synaptic location between an NG2 cell and an interneuron owing to the difficulty of distinguishing silver-enhanced double immunogold labelings within either the presynaptic NG2 terminal or the postsynaptic dendrite, respectively. Our present data do favor a tight inhibitory synaptic junctional complex between NG2 glia processes/terminals and interneurons based on their close spatial contact (Fig. 3e, f), GABA-immunogold labeled NG2 terminals (Fig. 3h), and the size of the inhibitory synaptic cleft domain they form^80,81^. Thus, it reveals an important role of NG2 glia fine-tuning of GABAergic neurons in a hippocampal microcircuit. Of interest was the observation that both NG2-positive and NG2-DAB/GABA-immunogold labeled nerve terminals were also making an asymmetrical synaptic contact onto a dendritic spine. It is highly unusual for a GABAergic-positive terminal of neuronal origin to make an asymmetrical synaptic contact onto a spine. Nevertheless, it has been occasionally observed that GABA-immunogold positive nerve terminals make an asymmetrical synaptic contact onto a dendrite in the hypothalamus^47^. Therefore, it would be interesting to elucidate accurately at the EM level, in future studies, as to whether the observed NG2/GABA asymmetrical synaptic contact is functionally excitatory and whether the spine originates from a pyramidal cell or an interneuron.

GABA, as well as glutamate, is one of the most prevalent neurotransmitters released mainly by inhibitory neurons and is crucial for maintaining brain network functionality. The change of GABA neurotransmitter level in the synaptic cleft can be mainly caused by: (1) l-glutamic acid decarboxylase (GAD) 65/67 biosynthesis; (2) glutamate/GABA-glutamine cycle; (3) GABA uptake through GABA transporters^49,50,82–84^. Our results indicate that the enhancement of mIPSCs frequency onto interneurons by NG2 glia photoactivation is not mainly regulated through a GABA transport mechanism (Supplementary Fig. 15)^16,85^. Instead, our data provide evidence that NG2 glia activation favors GABA signaling transmission through a GAD67 synthetase and VAMP-2-containing vesicular assembly machinery, thus enhancing the inhibitory synaptic activity directly onto local, adjacent GABAergic interneurons in the hippocampal CA1 region (Figs. 2–5). Therefore, our results presented in the study demonstrate that adult NG2 glia has the capability of releasing the neurotransmitter GABA, in addition to the secretion of growth factor FGF2^74^, indicating an additional source of GABA from NG2 glia besides inhibitory neurons in the brain.

Anxiety disorders are a category of mental illnesses with an estimated prevalence of ~7.3% of the world population characterized by feelings of anxiety and fear^86^. We hypothesize that altered GABA transmission and E-I imbalance largely contribute to the pathophysiology of anxiety disorders in humans and rodents as well^68,87^. In the hippocampus, complex spiking of pyramidal cells and their synchronized activity are under tight control by local inhibitory circuits within CA1^88–90^. For instance, NPY positive GABAergic neurons are able to integrate inputs from both the CA3 and the entorhinal cortex to trigger complex spikes in pyramidal cells in the Dp(16)1Yey model of Down Syndrome^37^. Here, our functional studies demonstrate that GABA released from NG2 glia impacts inhibitory synapses of proximal interneurons and reduces the GABA output from interneurons, which in turn enhances the excitability of pyramidal neurons, ultimately perturbing the E-I balance in adult hippocampal microcircuit to trigger an anxiety-like behavior (Figs. 6, 7). By utilizing GCaMP6s to visualize the calcium signals in NG2 glia, we found an intrinsic activation of NG2 glia when the mouse is exposed to CSDS, which is directly linked to anxiety-like behavior in CSDS. Taken together, our evidence from transcriptomic, ultrastructural, electrophysiological, and behavioral levels sheds light on a new potential target, NG2 glia, for the treatment of GABA-associated neurologic and anxiety-like neuropsychiatric disorders^91^.

## Methods

### Animals

All mouse experiments were approved by the Animal Ethics Committee of Shanghai Jiao Tong University School of Medicine (AAALAC accreditation Unit, 001670) and the Institutional Animal Care and Use Committee, the protocol number is A-2018-028. All mice were kept on a C57BL/6 background and under a standard condition with temperatures of 21–23°C, 40–60% humidity, and a 12 hr–12 hr light–dark cycle with food and water provided ad libitum from the cage lid.

Pdgfrα-creER^TM^ (JAX strain 018280, B6N.Cg-Tg(Pdgfra-cre/ERT)467Dbe/J), ChR2(H134R)-eYFP (Ai32) (JAX strain 024109, B6.Cg Gt(ROSA)26 Sortm32-(CAG-COP4*H134R/EYFP)Hze/J), Rosa26-mGFP (JAX strain 007676, mT/mG B6.129(Cg)-Gt(ROSA)26Sortm4(ACTB-tdTomato,-EGFP)Luo/J) mice were obtained from the Jackson Laboratory (USA). GCaMP6s^flox^ (JAX strain 024106, 129S6-Gt(ROSA)26S or Ai96) mice were a gift from professor Nanjie Xu, ROSA26iDTR (JAX strain 007900, Gt(ROSA)26Sortm1(HBEGF)Awai/J) mice were a gift from professor Qian Li, GAD67-GFP knock-in mice (RBRC03674, ICR.Cg-Gad1 < tm1.1Tama>)^92^ were a gift from professor Jiangteng Lv at Shanghai Jiao Tong University School of Medicine (Shanghai, China). NG2-creER^TM^ (JAX strain 008538, B6.Cg-Tg(Cspg4-cre/Esr1*)BAkik/J) was gifted from professor Chong Liu at Zhejiang University (Zhejiang, China). C57BL/6 J mice (JAX strain 000664) were obtained from the Slac Laboratory Animal (Shanghai, China). To induce Cre recombinase in Pdgfrα-creER^TM^; ChR2(H134R)-eYFP mice, Pdgfrα-creER^TM^; Rosa26-mGFP mice and NG2-creER^TM^; GCaMP6s mice, 120 mg/kg tamoxifen (ABCONE, T56488) dissolved in sunflower seed oil (SIGMA-ALDRICH) was intraperitoneally injected for 3–5 consecutive days.

### Acute brain slice preparation

For the preparation of brain slices, mice at postnatal 4–6 weeks were deeply anesthetized with 5% chloral hydrate and intracardially perfused with oxygenated ice-cold dissection buffer containing (in mM): 82.75 NaCl, 2.4 KCl, 6.8 MgCl_2_, 0.5 CaCl_2_, 1.4 NaH_2_PO_4_, 23.8 NaHCO_3_, 23.7 d-glucose, and 65 Sucrose. Coronal hippocampal slices were cut at 300 µm thickness (VT1000S; Leica Microsystems, Germany) and allowed to equilibrate for at least 1 hr at 31°C in aCSF containing (in mM): 125 NaCl, 2.5 KCl, 1 MgCl_2_, 2 CaCl_2_, 1.25 NaH_2_PO_4_, 25 NaHCO_3_, and 12.5 D-glucose. All the buffers in this experiment were continuously bubbled with a mixture of 95% O_2_/5% CO_2_ gas.

### Electrophysiological recordings from acute brain slices and cultured cells

For brain slice whole-cell patch recordings, an individual slice was placed in the recording chamber and continuously perfused with oxygenated aCSF at room temperature (RT). Slices were visualized with an upright epifluorescent microscope (BX51WI, Olympus, Tokyo, Japan) equipped with differential interference contrast optics and an infrared CCD camera (optiMOS, Q IMAGING, Olympus, Tokyo, Japan). All the electrophysiological recordings were made in the hippocampal CA1 region with a MultiClamp 700B amplifier (Molecular Devices, Sunnyvale, CA, USA). Signals were low-pass filtered at 2 kHz and sampled at 20 kHz using Digidata 1550 A (Molecular Devices) and data were collected 2 min after obtaining a stable whole-cell configuration. Patch pipettes were pulled from borosilicate glass capillaries with a microelectrode puller (Model P-1000, Sutter Instruments).

Photostimulation was performed using a blue collimated light-emitting diode (LED) with a peak wavelength of 470 nm (XT640-W, Lumen Dynamics) and the intensity of photostimulation was directly controlled by the stimulator (5~10 mW/mm^2^). The blue light was applied after at least 2 min of steady current was recorded. The slice was illuminated with 10 ms duration flashes at 15 Hz for 90 s through the 40 × 0.8 N.A. objective during the mIPSCs or mEPSCs recordings.

For NG2 glia and neuronal mEPSCs patch recordings, low chloride intracellular solution was used. It contained (in mM): 125 k-gluconate, 15 KCl, 8 NaCl, 10 HEPES, 0.2 EGTA, 3 Na_2_-ATP, and 0.3 Na-GTP (pH to 7.3). Cells were held at −80 mV and −70 mV in the voltage-clamp mode for NG2 glia and hippocampal neurons, respectively.

For hippocampal neurons mIPSCs patch recordings (including the dual-patch recordings), high chloride intracellular solution containing (in mM): 130 KCl, 2 MgCl_2_, 0.5 CaCl_2_, 2.5 Na_2_-ATP, 0.3 Na-GTP, 10 HEPES and 1 EGTA (pH to 7.3 with KOH) was used. Cells were held at −70 mV in voltage-clamp mode. In all, 20 μM DNQX, 50 μM D-AP5, and 1 μM TTX were added into the bath to block AMPA, NMDA, and Na^+^ channel currents, respectively, to isolate mIPSCs. In all, 20 μM bicuculline and 1 μM TTX were added into the bath to block GABA_A_Rs and Na^+^ channel currents to isolate mEPSCs. Tonic GABA_A_R-mediated current was defined as the steady-state current blocked by saturating concentrations of bicuculline, and its magnitude was calculated by plotting all-point histograms of relevant 20 s segments of data. The miniature IPSCs and EPSCs were analyzed with the Mini-Analysis 6.0 program by Synaptosoft. In some neuronal recordings, 20 µM Alexa Fluor 568 (Invitrogen, A10437) was added in the intracellular solutions for fluorescent dye injection and post-immunohistochemistry detection. Membrane access resistance of whole-cell patch recording was monitored before and after recording in all electrophysiological experiments and data with a > 20% change were excluded from the analysis. For sniffer patch, HEK-293T cells (ATCC, #CRL-3216) were digested and seeded in primary cultured NG2 cells after transfection with GABA_A_Rs-mCherry or control vector for 24 hr. Only GABA_A_Rs-mCherry or mCherry positive HEK-293T cells were whole-cell patches recorded while giving optogenetic stimulations in ChR2-expressing cultured NG2 cells. High chloride intracellular solutions (see brain slices whole-cell recordings) were used and external solutions contained (in mM): 150 NaCl, 10 Glucose, 10 HEPES, 2 CaCl_2_, 5 KCl, 1 MgCl_2_, pH adjusted to 7.3 with Tris-base.

### Immunohistochemistry and image analysis

Mice were anesthetized and perfused through the ascending aorta with a solution of normal saline for ~3 min, followed by 4% paraformaldehyde (PFA) in 0.1 M PB for 5 min. Brains were removed and post-fixed in 4% PFA at 4°C overnight and then cut into 40-µm-thick coronal sections including cortex and hippocampus. Slices were incubated in permeabilizing buffer (0.3% Triton X-100 in PBS) for 15 min and then blocked with donkey serum (Ruite Biotechnology, w9030-05) (5% in PBS-T: PBS with 0.1% Triton X-100) for 2 hr at RT. The primary antibodies included: rabbit antibody to NG2 (1:250, Millipore AB5320), Goat antibody to Pdgfrα (1:300, R&D Systems AF1062), rabbit antibody to GFAP (1:1500, Abcam ab7260), chicken antibody to GFP (1:500, Abcam ab13970), mouse antibody to Olig2 (1:500, Millipore MABN50), mouse antibody to CC1 (1:500, Millipore OP80), mouse antibody to cFos (1:1000, Abcam ab208942), mouse antibody to NeuN (1:500, Abcam ab104224), rabbit antibody to CCK-8 (1:1000, Sigma C2581), rabbit antibody to NPY (1:1000, Cell Signaling 11976 S), mouse antibody to PV (1:2000, Sigma P3088), rat antibody to SST (1:500, Millipore MAB354), guinea pig antibody to vGluT2 (1:200, Synaptic Systems 135404), rabbit antibody to VAMP-2 (1:1000, Alomone labs ANR-007), mouse antibody to Gephyrin (1:500, Synaptic Systems 147011), rabbit antibody to CaMKII (1:200, Abcam ab52476). The corresponding secondary antibodies included: Donkey anti-Rabbit Alexa Fluor 568 (1:500, Invitrogen A10042), Donkey anti-mouse Alexa Fluor 647 (1:500, Invitrogen A31571), Goat anti-Chicken Alexa Fluor 488 (1:500, Invitrogen A11039), Donkey anti-Goat Alexa Fluor 488 (1:500, Invitrogen A11055), Goat anti-Rat Alexa Fluor 647 (1:500, Invitrogen A21247), Goat anti-Rabbit Alexa Fluor 647 (1:500, Cell Signaling 4414 S), Goat anti-Rabbit Alexa Fluor 488 (1:500, Cell Signaling 4412 S), Goat anti-Guinea pig Alexa Fluor 647 (1:500, Invitrogen A21450). Sections were incubated with DAPI (1:1000, Cell Signaling 4083 S) for 15 min and mounted on glass slides in Fluoromount™ Aqueous Mounting Medium (AQUA-MOUNT, REF 13800). All images were acquired on a Leica TCS SP8 confocal microscope equipped with HC PL APO CS2 ×20/0.75 DRY, HC PL APO CS2 ×40/1.30 oil, or HC PL APO CS2 ×63/1.40 oil objective at the Core Facility of Basic Medical Sciences, Shanghai Jiao Tong University School of Medicine. Image analysis was performed by ImageJ 1.52a (US National Institutes of Health). The images of different channels were thresholded, cell numbers were determined according to the DAPI channel threshold image.

For 3D reconstruction of confocal images from Pdgfrα­creER^TM^; mGFP mice, the sections were immunostained with GFP (1:500, Abcam ab13970) and Gephyrin (1:500, Synaptic Systems 147011). The confocal Z stack images were captured every 0.6 μm consecutively on a Leica SP8 confocal microscope (Leica, Tokyo, Japan) with HC PL APO CS2 ×63/1.40 oil objective. The Z stack images were simulated to 3D movies using LAS X software (Leica Microsystems).

### RNAscope in situ hybridization

RNAscope Multiplex Fluorescent Reagent Kit v2 (Advanced Cell Diagnostics) was used following the manufacturer’s manual for fixed frozen tissues. In brief, Pdgfrα-creER^TM^; Rosa26-mGFP mice at postnatal 6–12 weeks were deeply anesthetized and perfused with freshly prepared 4% PFA. Brains were removed and post-fixed overnight at 4°C in the same fixative. After dehydration by gradient sucrose, brains were cut into 20-μm-thick sections on cryostat microtome (Leica, CM1950). Following RNAscope Hydrogen Peroxide incubation, antigen retrieval was performed using RNAscope Target Retrieval Reagent at 95–99°C for 5 min and RNAscope Protease III at 40°C for 30 min. RNAscope probes targeting *Gad1-C3* probe (Cat No.400951, ACD), *Slc17a7-C1* probe (Cat No.416631, ACD) were incubated on sections at 40°C for 2 hr. RNAscope 3-plex Positive control probe and 3-plex Negative control probe were used as positive and negative controls, respectively. The slides were processed for standard signal amplification steps. Fluorescent detection was performed using TSA Plus Cy5 and Cy3 following TSA Plus System instructions (PerkinElmer). Sections were counterstained with DAPI for 30 s at RT and mounted on glass slides in Fluoromount™ Aqueous Mounting Medium (AQUA-MOUNT, REF 13800).

As for IHC post-RNAscope ISH, after in situ hybridization, sections were directly immunostained. *Gad1-C3* probe (Cat No.400951, ACD), *Slc17a7-C1* probe (Cat No.416631, ACD), and GFP antibody (1:300, Abcam, ab13970) were used and images were acquired on a Leica TCS SP8 confocal microscope (Leica, Tokyo, Japan) with HC PL APO CS2 ×63/1.40 oil objective at the Core Facility of Basic Medical Sciences, Shanghai Jiao Tong University School of Medicine.

### NG2 glia and interneurons isolation by FACS

The Pdgfrα-creER^TM^;Rosa26-mGFP or GAD67-GFP mice were used to purify NG2 glia or interneurons by FACS. The brain tissues at postnatal days 21–28 were dissociated following published guidelines with slight modifications^11^. In brief, mice were anesthetized and intracardially perfused with ice-cold carbonated (95% O_2_, 5% CO_2_) aCSF containing: 125 mM NaCl, 2.5 mM KCl, 1 mM MgCl_2_, 2 mM CaCl_2_, 1.25 mM NaH_2_PO_4_, 25 mM NaHCO_3_, and 12.5 mM d-glucose. Then the brain was removed and coronal sections of the brain were cut at 300 µm thickness (VT1200S, Leica Microsystems, Germany) and allowed to equilibrate for 30 min at 31°C in aCSF, which was continuously bubbled with a mixture of 95% O_2_/5% CO_2_ gas. After that, the brain slices were digested for 45 min at 37˚C in 50 ml centrifuge tubes with 3 ml papain solution (15 U/ml papain, 75 U/ml DNase I, 2 mM cysteine, 50 mM EDTA). Thereafter, the digestion was stopped by protease inhibitor solution (1 mg/ml ovomucoid, 0.1% BSA, and 75 U/ml DNase I). The tissue was immediately triturated and centrifuged at 4°C at 300 × *g* for 3 min. The pellet was resuspended in D-PBS with 0.1% BSA and filtered with a 70 µm mesh. FACS was performed in a Beckman Coulter Moflo Astrios with a 70 µm nozzle using standard methods at the Core Facility of Basic Medical Sciences, Shanghai Jiao Tong University School of Medicine. The sorted cells were analyzed with Summit software and used for subsequent experiments.

### Transcriptomic analysis

For bulk RNA-seq transcriptome experiment, the cDNA from NG2 glia was synthesized and amplified from ~10,000 cells using SMART-seq™ v4 ultra-low input RNA kit (Takara, Mountain View, CA) to generate double-stranded cDNA for each replicate following the manufacturer’s instructions. Illumina libraries were prepared using the commercial Sample Preparation kit (Nextera XT DNA Library Prep Kit) according to the manufacturer’s instructions. The barcoded single-cell Illumina libraries of each experiment were pooled and sequenced for 2 × 75-base Paired-End reads on Illumina NextSeq500 sequencing system at the Sequencing Core of Shanghai Institute of Immunology, Shanghai Jiao Tong University School of Medicine. Sequencing reads were inspected by Fastqc 0.11.3 to check the quality of the read and then aligned to the GRCm38/mm10 assembly of the mouse genome using Tophat 2.1.0 with the default options. FPKM (fragments per kilobase of exon per million fragments) values of each gene were obtained by Cufflinks 2.2.1 using genome annotation from UCSC (University of California, Santa Cruz). The Log2 (FPKM) values of the genes involved in GABA and glutamate synthesis and transport, and SNARE complex were plotted as heatmaps, and the vesicle-associated membrane proteins—encoded genes were plotted as a pie chart. RNA-sequencing data are deposited at the Sequence Read Archive with SRA accession number SRP215327.

For single-cell RNA-seq experiment, Chromium Single Cell 3′ v3 (10× Genomics) library preparation was conducted by the Sequencing Core at the Shanghai Institute of Immunology according to the manufacturer’s instructions. The resulting libraries were sequenced with an Illumina novaSeq 6000 platform. The Cell Ranger software pipeline (version 3.0.0) provided by 10× Genomics was used to demultiplex cellular barcodes, map reads to the mm10 reference assembly (v1.2.0, 10× Genomics), and generate gene UMI (unique molecular identifier) counts versus cells barcode matrix. The Seurat R package (version 3.2.0) was used for further inspection and data analysis^93^. The resulting filtered matrix consisted of 17,753 genes and 4,014 cells. The matrix was normalized using the NormalizeData() function and variable features were identified using FindVariableFeatures() with 2000 genes. The ScaleData() function was used to center the gene expression. Next, principal component analysis (PCA) was performed, using RunPCA() function, to obtain the top 50 principal components (PCs). Clustering was conducted using the FindNeighbors() and FindClusters() functions using 20 PCs and a resolution parameter set to 0.3. For visualization, the dimensionality of the data sets was reduced by t-SNE, using the RunTSNE() function in the Seurat package. Cell populations were matched to cell types based on the expression of known marker genes and previously identified expression signatures^94^. Single-cell RNA-seq data are available in GEO (accession number: GSE162049).

### Single-cell RT-PCR and western blotting

Single GFP(+) NG2 glial cells or GFP(−) interneurons from Pdgfrα-creER^TM^;mGFP mice at postnatal 6 weeks were selected and aspirated into a glass pipette from hippocampal acute slices following a method described previously^11^. In brief, cells were grabbed promptly by micromanipulation and immediately placed in lysis buffer. To minimize potential changes in gene expression, all cells were collected within 3 hr after slice preparation. Selected cells were processed for single-cell RNA extraction and reverse transcription within 1 hr and were subjected to cDNA amplification and purification. Single-cell cDNA was amplified using KAPA HiFi HotStart ReadyMix (2×; KAPA Biosystems, Cat. No. KK2601) according to the manufacturer’s protocol^95^. PCR was performed by using specific primers targeting *Gad1*, *Gad2*, *Pdgfrα*, *Egfp*, and *Gapdh* as listed in Supplementary Table 1. *Gapdh* was used as an internal control.

For evaluation of GAD67 protein expression by western blot analysis, the sorted NG2 glia, MOLT-4 cells (ATCC, #CRL-1582), and C57BL/6 mice hippocampus were collected and lysed in buffer containing: 50 mM Tris HCl, 150 mM NaCl, 1% NP-40, 0.5% deoxycholate, 0.1% SDS, protease inhibitors cocktail (Cat. No. 04 693 132001; Roche), pH 7.6 and centrifuged at 12,000 × *g* for 20 min at 4°C. The supernatants were measured with a BCA kit (Thermo Fisher Scientific, USA). The supernatants were denatured at 95˚C for 10 min and then the proteins were separated on a Tris-glycine gel and transferred into a polyvinylidene difluoride (PVDF) membrane (Millipore, USA). Membranes were blocked with 5% BSA in 0.01 M Tris-buffered saline (pH 7.4) containing 0.1% Tween-20 (TBST) for 2 hr at RT. PVDF membranes were incubated with antibodies against GAD67 (Millipore, USA) or GAPDH (Sangon, China) at 4°C overnight. After washing three times with TBST, the membrane was incubated with HRP-conjugated goat anti-mouse IgG at RT for 2 hr. Protein bands were visualized by enhanced chemiluminescence reagent (Thermo Fisher Scientific, USA). For quantification, the optical density of the gel bands was determined using ImageJ (US National Institutes of Health).

### Immunoelectron microscopy and data analysis

For silver-enhanced immunogold labeling, in brief, C57BL/6 mice at postnatal 6–8 weeks were deeply anesthetized with 10% chloral hydrate intraperitoneally and perfused transcardially with saline, followed by ice-cold mixture of 2% PFA and 0.1% glutaraldehyde (GA) in 0.1 M PB (pH 7.4). Brains were dissected and post-fixed for 4 hr by immersion in the same fixative at 4°C. Coronal vibratome (Leica, VT1000S) free-floating sections of 50 μm thickness were collected in 0.1 M PBS. Sections were immersed in 50 mM glycine (in PB) for 30 min, then blocked with blocking solution (5% BSA, 5% NGS, and 0.05% Triton X-100 in PBS) for 2 hr, and incubated with anti-NG2 (1:75, Millipore, AB5320) antibody diluted with a solution containing 1% BSA, 1% NGS and 0.05% Triton X-100 for 24–48 hr at 4°C. After rinsing, sections were incubated with 1.4-nm gold-conjugated anti-rabbit IgG (1:100, Nanoprobes) for 12–24 hr at 4°C. After rinsing, sections were post-fixed in 2.5% GA in PB for 2.5 hr. Silver enhancement was performed in the dark with the HQ Silver Kit (Nanoprobes), as directed by the manufacturer. Before and after the silver enhancement step, sections were rinsed several times with ddH_2_O. Immunolabelled sections were post-fixed with 1% osmium tetroxide in PB for 1 hr and then incubated in 2% uranyl acetate in ddH_2_O for 40 min in the dark. Sections were dehydrated in graded ethanol, then acetone series, and finally flat-embedded in Epon 812. After polymerization, flat-embedded sections were examined under a light microscope. Serial ultrathin (~70–90 nm) sections were cut with an Ultramicrotome (Leica EM UC6, Germany) using a diamond knife (Diatome) and mounted on formvar-coated mesh grids (6–8 sections per grid). They were observed under a Tecnai G2 Spirit 120 kV transmission electron microscopy at the Center of Cyro-Electron Microscopy, Zhejiang University.

Random sections from the pre-embedding immunogold labeling specimen above were imaged using TEM. EM images were taken at ×30,000 with a field of view (FOV) of 11.21 µm^2^. In all, 41 FOVs from four C57BL/6 mice were collected and analyzed. To exclude non-specific immunogold labeling, the determination of a presynaptic NG2 glial processes/terminal, making a synaptic contact onto a postsynaptic neuronal structure was recognized with the following criteria: (1) dark and dense immunogold particles (diameter >10 nm) labeled with three or more along the plasma membrane; (2) presence of synaptic vesicles; (3) visually apparent synaptic cleft; and (4) identification of a postsynaptic density^40,41^.

For DAB staining combined with GABA-immunogold EM, which was performed as previously described^45,96^. In particular, in order for the post-embedded GABA-immunogold labeling to work successfully after first carrying out NG2-DAB staining, we have to use a more concentrated 2.5% GA fixative instead of the more conventional 2% PFA fixation of the tissue since the specific GABA antibody is made against a GABA-GA conjugate. This unique procedure is critical for the successful expression of GABA-immunogold particles located within NG2-DAB-positive nerve terminals. In brief, C57BL/6 mice (*n* = 5) at postnatal 8 weeks were anesthetized with mouse cocktail (0.2% ketamine, 0.02% xylazine in normal saline) and perfused using a transcardiac approach with 6 ml of heparin (1000 units/ml) in 0.1 M PB (pH 7.4), followed by 50 ml of electron microscopy (EM) fixative [2.5% GA, 0.5% PFA, and 0.1% picric acid (PA) in 0.1 M PB]. Brains were removed, cut in half coronally at the level of the hippocampus, both halves were then placed in EM fixative and further fixed in a microwave tissue processor (Pelco BioWave, Ted Pella), containing a temperature-controlled fixation bath using a thermoelectric recirculating chiller (Pelco Steady Temp Pro, Ted Pella) for a total of 30 min [150 watts (W) for 20 min at 28°C, 650 W for 10 min at 25°C]. Brain halves were then rinsed and left in 0.1 M PB at 4°C until serially sectioned through the dorsal hippocampus at 60 µm using a vibratome (Leica, Germany). Pre-embed IHC of the dorsal hippocampus using DAB (Sigma, D5637) immunolabeling using an anti-NG2 antibody (1:75, Millipore, AB5320). After the incubation with the secondary antibody (biotinylated goat anti-rabbit, 1:50, Jackson ImmunoReseach, 111-065-003), the slices containing the dorsal hippocampus was reacted with DAB and further processed as previously described^45,96^. Following NG2-DAB labeling processing, 2–3 NG2-labeled hippocampal sections were then mounted on gelatin-coated slides, cover-slipped, and examined to determine the localization of the DAB reaction product within the CA1 region. The remaining tissue was prepared for EM and the dorsal hippocampus was microdissected out of the embedded tissue and superglued onto a separate block for each area/animal. The tissue was then thin-sectioned and post-embed immunogold labeling, using a primary antibody anti-GABA (non-affinity purified, rabbit polyclonal, 1:250, Sigma, A2052) and 12 nm gold-conjugated anti-rabbit IgG (Jackson ImmunoReseach, 1:50, 111-205-144), which was diluted in TBST (Tris-buffered saline with Triton X-100, pH 7.6) in blocking solution (0.5% BSA, Electron Microscopy Sciences) and diluted in TBST, pH 8.2 respectively. One thin section was placed on a formvar-coated nickel slot grid (Electron Microscopy Sciences). Following immunogold labeling of the thin-sectioned section, they were counterstained with both uranyl acetate and lead citrate. Pre-incubation of the antibody with 5 mM GABA to the thin-sectioned tissue, resulted in no immunogold labeling^45^. Using a JEM-1400 transmission electron microscope, photographs were randomly taken in DAB-labeled areas of the CA1 region (at the leading edge of the tissue section). The DAB reaction product can be recognized as “patchy/cloudy” signals.

### Primary NG2 glia/OPCs culture and plasmid transfection

As for the NG2 glia culture^97,98^, mouse cortices including hippocampus were isolated from Pdgfrα-creER^TM^; ChR2-eYFP pups at postnatal day 1. Cortical tissues were diced into ~1 mm^3^ pieces in a 60 mm dish with a sterilized razor blade. The minced tissues were transferred to digestion solution [(trypsin solution, 0.25% (Gibco, Baltimore, MD); Dnase I, 75 U/ml (Worthington, Lakewood, NJ)] and incubated for 10 min in the tissue culture incubator at 37°C. The cells were collected by centrifugation in a swinging bucket at 1000 × *g* for 5 min. The pellet was resuspended with freshly prepared ice-cold neurosphere growth medium (Dulbecco’s Modified Eagle Medium (DMEM)/F12 supplemented with B27 (Gibco, Baltimore, MD) and 10 ng/ml EGF (Peprotech, Rocky Hill, NJ)) and added 5 × 10^5^/ml cells to the dishes. Half of the medium was replaced with a fresh neurosphere growth medium for 8–10 days every 2 days. After the neurospheres formed, the EGF containing neurosphere growth medium was changed to oligosphere medium (DMEM/F12 supplemented with B27, 10 ng/ml platelet-derived growth factor (PDGF, Peprotech, Rocky Hill, NJ), 10 ng/ml bFGF (Peprotech, Rocky Hill, NJ) and 1 µM (Z)-4-OHT ((Z)-4-Hydroxytamoxifen, SIGMA-ALDRICH)). After the oligospheres formed for 7–9 days, the spheres were continuously digested by trypLE (Gibco, Baltimore, MD) and OPCs/NG2 cells were plated at 5 × 10^5^/ml on a new PDL-coated dish in OPC medium [(DMEM/F12 supplemented with B27, N2 (Gibco, Baltimore, MD), 0.1% BSA, 10 ng/ml PDGF, 20 ng/ml bFGF, 5 µg/ml IGF (Peprotech, Rocky Hill, NJ) and 1 µM (Z)-4-OHT)].

For the GABA_A_Rs transfection, we co-transfected the plasmids GABARα1, GABARβ2, and GABARγ2-mCherry into HEK-293T cells with Lipofectamine 2000 reagent (Thermo Fisher Scientific Inc., Waltham, MA, USA) according to manufacturer’s instructions. The plasmid GABARγ2-mCherry was obtained by subcloning the *Gabrg2* gene into the vector pLVX-IRES-mCherry using the XhoI and BamHI restriction sites. For the VAMP2-pHuji plasmid, we made this construct with a slight modification^99^. In brief, we first replaced the EGFP of the vector pEGFP-N1 with pHuji which was synthesized by Sangon Biotech (China), to yield ppHuji-N1 vector. Then we cloned the *Vamp2* gene to insert into the ppHuji-N1 between the EcoRI and BamHI sites. The modified construct was verified by sequencing before the transfection. VAMP2-pHuji plasmid or pGEMTEZ-TeTxLC plasmid^100^ was diluted at the calculated concentration by OPTIM-MEM (Gibco, MA, USA) and the NG2 cells were transfected by a mixed medium in an incubator for 6 hr before it changed back in OPC culture medium. After 24 hr transfection, the cells were used for subsequent TIRFM imaging.

### Synaptosomes preparation and GABA content examination by HPLC

Synaptosomes were isolated and prepared as described previously^101^ with minor modifications. In brief, we carefully decanted the culture medium from cells and then washed the cells twice with ice-cold PBS. Added appropriate amount of sucrose buffer (0.32 M sucrose, 10 mM HEPES, 1 mM EDTA, and protease inhibitor cocktail, pH 7.5) to the plate. Scraped the plate surface using a cell scraper to lift the cells. After that, we collected the lysate and transferred it to a microcentrifuge tube. The cell lysate was centrifuged for 10 min at 1000 × *g*, and the supernatant was then layered on the top of a discontinuous sucrose gradient (0.8 M, 1.2 M) and centrifuged at 160,000 × *g* (Beckman, Optima XPN100) for 30 min. The final synaptosomal fraction was collected from the interphase between the 0.8 M and 1.2 M sucrose layers. Total protein in synaptosomes was quantified using the BCA Protein Assay Kit (Thermo Fisher Scientific, 23225).

To determine the GABA content, cells or isolated synaptosomes were sonicated in HClO_4_ lysis solution (0.2 N), and the supernatants were collected after centrifugation (12,000 × *g*) at 4°C for 20 min. HPLC (high-performance liquid chromatography) analysis was performed using the Agilent 1260 series neurotransmitter analyzer (Agilent Technologies, Santa Clara, CA, USA) which consists of a G1329B autosampler, a G1311B pump, and a G1312B fluorescence detector, at the Institute of Brain Science and the Collaborative Innovation Center for Brain Science, Fudan University. The sample’s supernatants were injected into an Eclipse XDB C18 column (5 μm, 4.6 × 250 mm; Agilent Technologies) at 24°C. Separations were performed at a flow rate of 0.8 ml/min using a mobile phase A consisting of 0.1 M KH_2_PO_4_, methanol, and tetrahydrofuran (volume ratio was 65:35:2). Phase B was 90% methanol. For the in-needle derivation, a 13 μl sample was aspirated followed by 7 μl of OPA reagent, then mixing took place three times with 2 min intervals. After the above steps, the mixture of 20 μl was injected into the LC system for analysis. The total acquisition time was 20 min. A step gradient was programmed switching from 100% solvent A to 40% solvent B within 10.9 min. At 13.5 min, 100% solvent B was applied and maintained until 16 min elapsed. At 18 min 100% solvent was applied again and the system was allowed to equilibrate during the derivation and injection. The excitation wavelength was 340 nm and the emission wavelength was 420 nm. The data were collected and analyzed by ChemStation (Agilent Technologies). Peaks and relative concentrations were identified by comparison to known external standards.

### TIRFM imaging

Imaging and analysis for TIRFM were performed as described previously^57,58^. In brief, an Olympus IX-83 inverted microscope equipped with a UAPON ×100 OTIRF N.A. 1.49 oil objective (Olympus, Japan) was used to observe the VAMP2-pHuji transfected NG2 cells cultured on a high-refractive-index glass coverslip and the images were captured by Prime95B CMOS camera (pixel size: 0.11 μm, Teledyne photometrics) with an exposure time of 50 ms. The setup consisted of an argon-ion laser (488 nm) and an argon-ion laser (561 nm) to detect VAMP2-pHuji and ChR2-eYFP-positive NG2 cells. All TIRFM experiments were performed at 37°C maintained by Microscope Incubator (H201, Oko lab). The cell bath solution contained (in mM): 150 NaCl, 10 Glucose, 10 HEPES, 2 CaCl_2_, 5 KCl, 1 MgCl_2_, pH to 7.3. We adjusted the camera gain and FOV for each NG2 cell to provide the best signal-to-noise ratio images. The event frequency between NG2 cells was reported as events number/second/10 × 10 µm^2^. An exocytotic event was defined as an abrupt fluorescence increase immediately followed by a decrease or diffusion of the fluorescence to the vicinity^102^. Spots larger than 100 pixels or less than four pixels were discarded.

### Calcium imaging and analysis

Primary cultured NG2 cells were incubated with 5 μM Rhod2-AM (Invitrogen, R1244) for 30 min in culture medium at 37°C incubator temperature and then washed twice and equilibrated for 30 min before experiments. Images were acquired every 1 s using an Olympus IX-83 inverted microscope equipped with a ×60 oil objective with 1.49 N.A. (Olympus, Japan) and a LED (Lumencor Spectra X) at a low light intensity (<1% power) to avoid possible fluorescence bleaching. Experiments were performed at RT in conventional extracellular solution containing (in mM): 150 NaCl, 10 Glucose, 10 HEPES, 2 CaCl_2_, 5 KCl, 1 MgCl_2_, pH at 7.3.

Slice imaging was performed using a two-photon microscope (Fluoview FVMPE-RS; Olympus, Japan) and a ×25, 1.05 N.A. water-immersion objective (Olympus, Japan) at the Core Facility of Basic Medical Sciences, Shanghai Jiao Tong University School of Medicine. The excitation wavelength was set to 920 nm and the emitted light was filtered to collect green light from GCaMP6s ([Ca^2+^]_i_). X-y time series had a frame size of 256 × 256 pixels (0.994 μm per pixel) and were acquired every 550 ms. Z series was set to a total 20 µm thickness (three z steps) to obtain the best cell imaging. Data were analyzed using GECI quant method as previously reported^103^. In brief, background subtraction was made for each time-lapse movie, and then polygonal regions of interest (ROIs) were placed around most of the visible NG2 glial cells’ somata and processes. For each ROI, basal F was determined during 60 sec periods with no fluctuations, and the final mean fluorescence intensity was expressed as dF/F to determine the size of the detected [Ca^2+^]_i_ transients.

### Optical fiber implantation

Adult Pdgfrα-creER^TM^; ChR2(H134R)-eYFP mice and their littermates (8–12 weeks old) were deeply anesthetized with 5% chloral hydrate via intraperitoneal injection and mounted on a stereotaxic device (Stoelting Co., IL, USA). The skull was exposed under aseptic conditions and a craniotomy was made bilaterally with a dental drill over dorsal CA1 (AP: −2.0; ML: ±1.3; DV: −1.6; mm relative to bregma). The optical fibers (diameter: 200 μm; N.A., 0.5; length, 2.5 mm; Inper, Hangzhou, China) were implanted into the target coordinates and fixed to the skull with screws and dental cement. After 1 week of recovery time, these mice were injected with tamoxifen for 5 consecutive days before the behavioral tests were performed. Fiber implantation sites were confirmed post hoc in all animals. Only mice with the correct location of optical fibers and ChR2 expression were used for further analysis.

### Open field test

The OFT has been widely used to evaluate the locomotor activity and anxiety-like behaviors in an open-field arena^104^. All experimental mice were transferred to the behavioral testing room at least 1 hr before the tests to reduce stress and to acclimatize to the environment. The test chamber (40 × 40 × 35 cm) was made of gray plastic, which was divided into a central field (center, 20 × 20 cm) and an outer field (periphery). Mice were individually placed in the center area of the chamber, and their paths were recorded by a video camera. To examine the effect of photostimulation of NG2 glia, the animals were tested during 25-min sessions (consisting of 5 min light off-on-off-on-off periods). Blue light (470 nm, 8–10 mW, 10 sec ON/OFF) was generated by an external laser power source (Newdoon Inc., Hangzhou, China) and delivered bilaterally during the light-on phase. Distance traveled and time spent in the center zone were analyzed with EthoVision XT 14 video tracking system (Noldus Information Technology, Wageningen, Netherlands). After each trial, the chamber was cleaned with 75% alcohol solution.

### Elevated plus maze test

Unconditioned anxiety-like behaviors were monitored using an EPM with two open arms (30 × 7 × 0.25 cm) and two closed arms (30 × 7 × 15 cm) made of opaque gray plastic and the EPM was elevated at a 60 cm height above the floor. Mice were placed in the central area of the apparatus with their heads facing one of the two open arms and allowed to explore for a 15-min session (consisting of 5 min light off-on-off period). The blue light was delivered as described in OFT method section and animal movement and location were recorded with EthoVision XT 14 video tracking system (Noldus Information Technology, Wageningen, Netherlands). Automated tracking was used for the analysis of time spent and the number of entries to the open and closed arms. All apparatuses and testing chambers were cleaned with 75% alcohol between animal tests.

### CSDS mouse model

CSDS was performed as described previously^64^. In brief, before the experiment, retired male CD1 breeders were screened on 3 consecutive days to validate their aggressiveness. Over the following 10 consecutive days, each experimental male NG2-creER^TM^; GCaMP6s mouse (intruder) was introduced into the home cage of a novel aggressive CD1 mouse (resident) for 10 min and was then physically defeated. After 10 min of physical interaction, intruders and residents were maintained in sensory contact for 24 hr using a perforated Plexiglass partition dividing the resident home cage into two halves. After 10 days of CSDS, experimental animals were housed singly. Control animals were housed in pairs, one on each side of a perforated Plexiglass partition, and they were never in physical or sensory contact with CD1 mice. All animals were tested for the OFT (25-min session) and the EPM (15-min session) as described above as both pre- and post-CSDS.

To examine the effect of photostimulation of NG2 glia on social avoidance elicited by social defeat, the procedure was identical to the normal CSDS procedure above, with the exception that adult Pdgfrα-creER^TM^; ChR2 (H134R)-eYFP mice and their littermates (8–12 weeks old) implanted with optical fibers as described above were used. After 10 days of CSDS, animals were housed singly and tested 24 hr later for social avoidance behavior.

### Social interaction

In brief, social avoidance behavior was measured according to a two-stage social interaction test. In the first stage, mice were placed in the open field arena containing a wire cup (8 cm diameter, 20 cm height) at one end of the arena. Time spent in the area surrounding the cup (interaction zone, an 8 cm region surrounding the cup) and in the corner area along the wall opposite the cup (corner zone, two 8 ×  8 cm^2^ corner regions along the wall opposite the cup) were measured individually. The time spent in the interaction zone in the first stage was termed “No target”. Animals were then returned to the home cage for 1 min. In the second stage, an unfamiliar, aggressive CD1 mouse was confined within the cup and the same metrics were measured (termed “Target”). From these two stages, the social interaction ratio was calculated (time spent in interaction zone in “Target”/time spent in interaction zone in “No target”). In all behavioral experiments except for non-CSDS control mice, blue light was delivered as described in OFT method section and the mice’ tracks were monitored with EthoVision XT 14 video tracking system (Noldus Information Technology, Wageningen, Netherlands). The arena and wire cup were cleaned with 75% alcohol between tests.

### Statistics and reproducibility

All experiments were performed with at least three biological replicates independently as indicated in the figure legends. All statistical tests were run in GraphPad InStat 3. The graphs were created in Origin 8 and assembled in CorelDraw 12. Data are presented as mean ± SEM. For each set of data to be compared, we determined in GraphPad Instat whether the data were normally distributed or not. If they were normally distributed, we used parametric tests. Paired and unpaired Student’s two-tailed *t* tests were used as appropriate and as indicated in each figure legend. If the data were not normally distributed, we used non-parametric tests. Two-tailed Mann–Whitney test or Tukey–Kramer multiple comparisons test were used as appropriate and as indicated in each figure legend. For electrophysiological experiments, *n* values represent the number of recorded cells. For all biochemistry, *n* values represent the number of mice. For immunohistochemistry, TIRF imaging, and calcium imaging experiments, *n* values represent the number of brain slices, cells, or vesicular particle numbers. For behavioral experiments, *n* values represent the number of tested mice. Investigators were blind to the groups or samples during the experiments. No statistical methods were used to pre-determine sample size, or to randomize. Statistical significance was set at *P < 0.05, **P < 0.01, ***P < 0.001.

### Reporting summary

Further information on research design is available in the Nature Research Reporting Summary linked to this article.

## Supplementary information

Supplementary Information

Reporting Summary

Peer Review File

Description of Additional Supplementary Files

Supplementary Movie 1

Supplementary Movie 2

Supplementary Movie 3

Supplementary Movie 4

Supplementary Movie 5

## Data Availability

The data supporting the findings of this study are included within the article and its Supplemental files. The accession number for the bulk and single-cell RNA-seq data is SRP215327 in SRA (Sequence Read Archive) and GSE162049 in GEO (Gene Expression Omnibus), respectively. Source data are provided with this paper.

## Source data

Source Data
