# Supplementary material for: NG2 glia-derived GABA release tunes inhibitory synapses and contributes to stress-induced anxiety
Source: Nat Commun. 2021 Sep 30;12:5740. doi: 10.1038/s41467-021-25956-y (PMC8484468; doi:10.1038/s41467-021-25956-y)
Supplement: Supplementary file 1 — Supplementary Information [file 41467_2021_25956_MOESM1_ESM.pdf]

Supplementary Figure 1

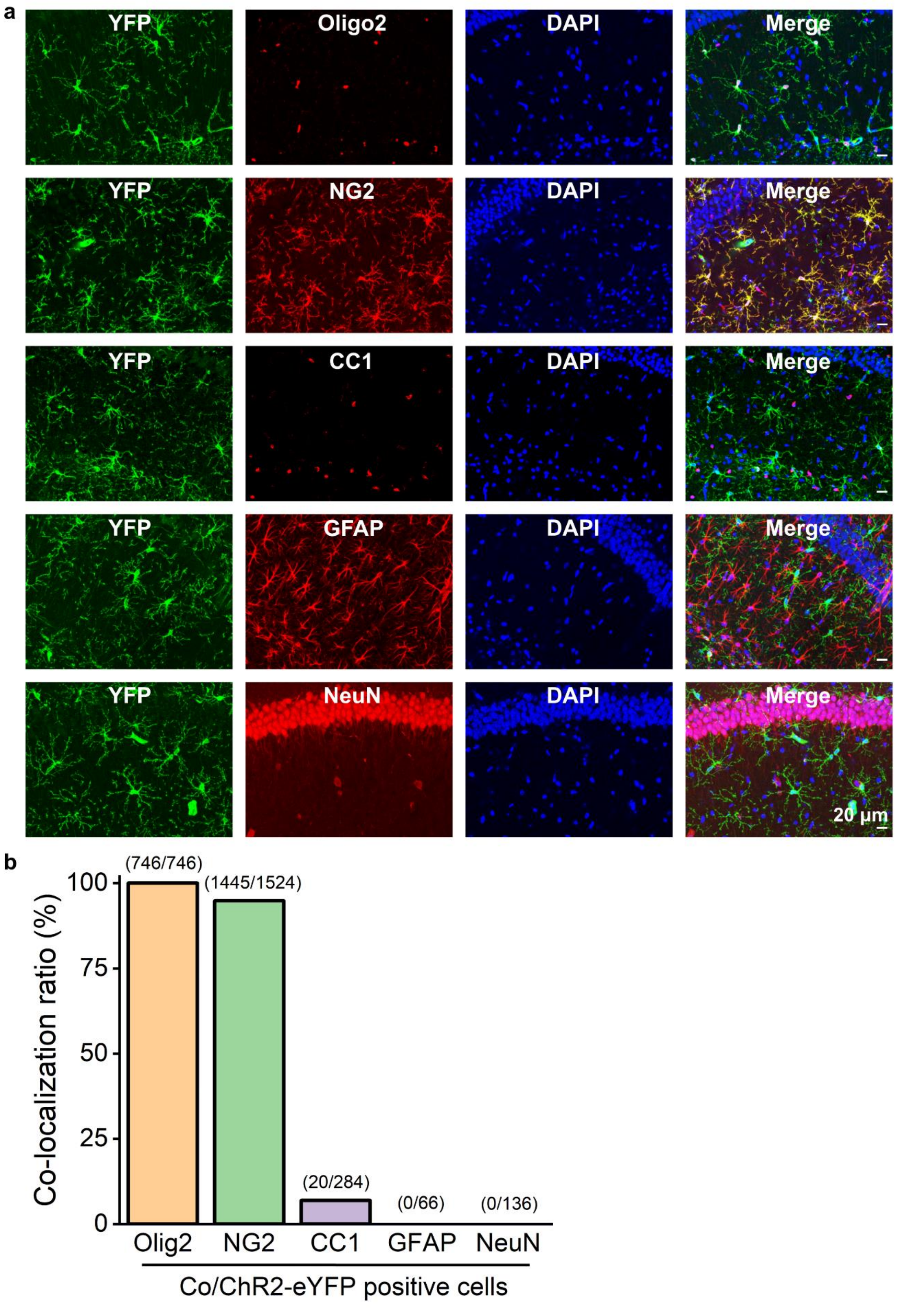

Supplementary Fig. 1 NG2 glia expression in Pdgfrα-creER^{TM}; ChR2-eYFP mice at postnatal 4-6 weeks. Representative images of immunohistochemistry (**a**) and summary bar graph (**b**) showing a 100% (n = 746 cells from 7 mice) colocalization between YFP-labeled cells and Olig2 (Oligodendrocyte lineage cells marker), a 94.69 ± 0.89% (n = 1524 cells from 12 mice) colocalization between YFP-labeled cells and NG2 antibody and a 6.93 ± 1.62% (n = 284 cells from 3 mice) colocalization between YFP-labeled cells and mature oligodendrocyte marker CC1, but was not immunostained with astrocytic marker GFAP (n = 66 cells from 1 mouse) nor with neuronal marker NeuN (n = 136 cells from 2 mice) in the hippocampus. Scale bars, 20 µm.

Supplementary Figure 2

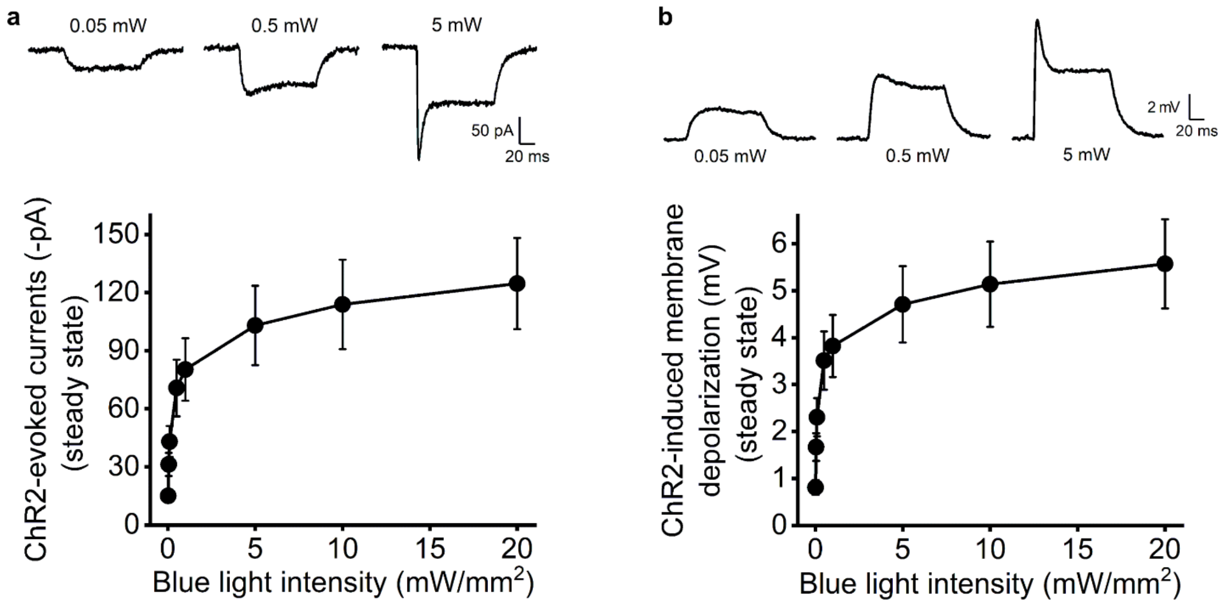

Supplementary Fig. 2 ChR2-evoked photocurrents and ChR2-induced membrane depolarizations in NG2 glia in Pdgfrα-creER^{TM};ChR2-eYFP mice at postnatal 4-6 weeks. **a**, Representative traces in the upper panel show blue light stimulation-induced photocurrents in NG2 glia at the intensity of 0.05, 0.5 and 5 mW/mm^2 illumination, respectively. Line chart in the lower panel summarizes ChR2-evoked photocurrents in NG2 glia with an intensity of 0.02, 0.05, 0.1, 0.5, 1, 5, 10 and 20 mW/mm^2 illumination, respectively. n = 8 cells recorded. **b**, Representative traces in the upper panel show blue light stimulation-induced membrane depolarizations in NG2 glia at the intensity of 0.05, 0.5 and 5 mW/mm^2 illumination, respectively. Line chart in the lower panel summarizes ChR2-induced membrane depolarizations in NG2 glia at the intensity of 0.02, 0.05, 0.1, 0.5, 1, 5, 10 and 20 mW/mm^2 illumination, respectively. n = 10 cells recorded. Data are presented as mean values ± SEM and error bar represents SEM.

Supplementary Figure 3

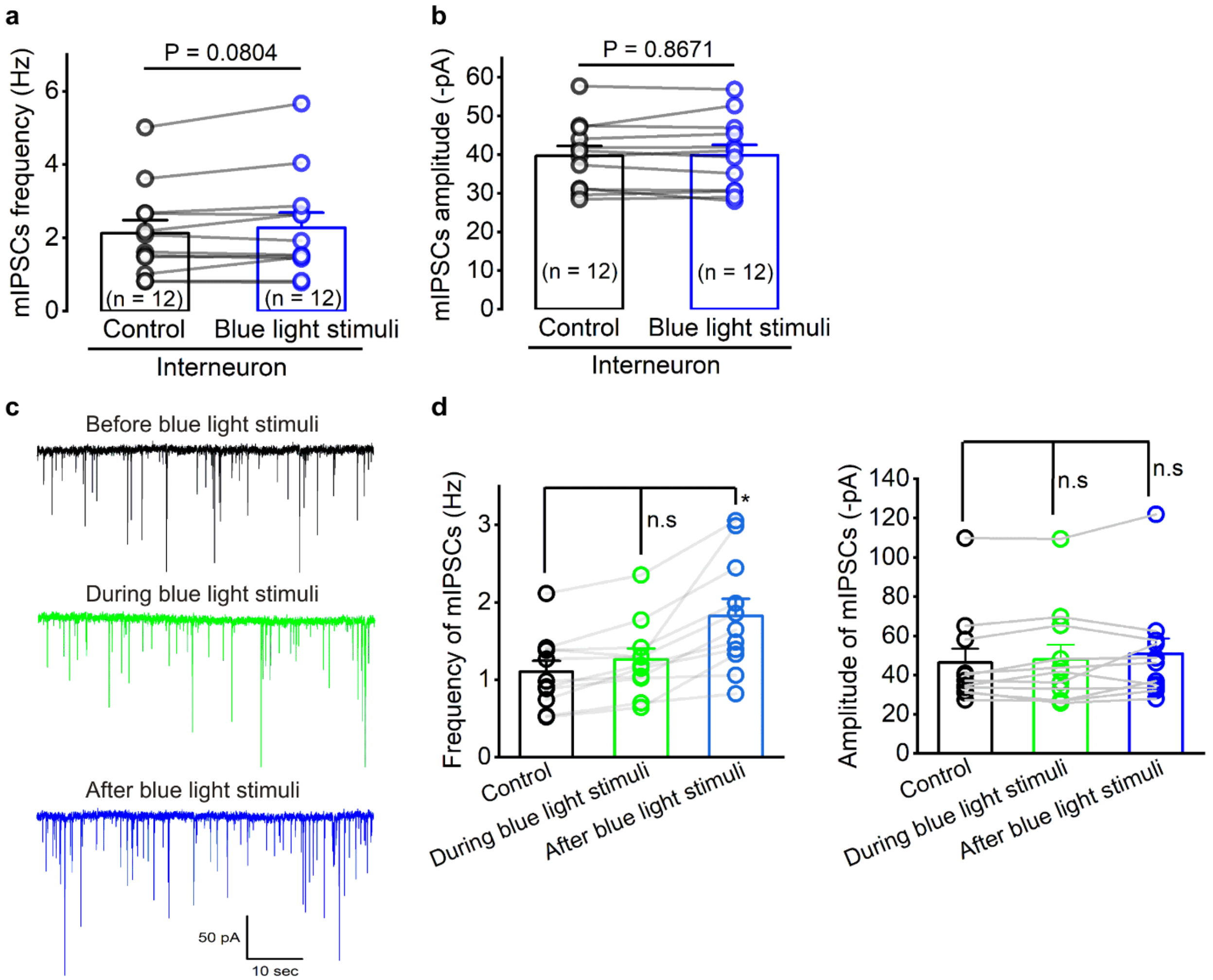

Supplementary Fig. 3 The effect of NG2 glia photoactivation on mIPSCs of interneurons in hippocampal CA1 region with different intensities of blue light illumination. **a-b**, Bar graphs show that there is no change of frequency or amplitude of miniature inhibitory postsynaptic currents (mIPSCs) in interneurons when NG2 glia are photostimulated at 0.5 mW/mm^2 illumination intensity. n = 12 cells from 4 mice at postnatal 4-6 weeks, two-tailed paired t-test. **c-d**, Representative traces of mIPSCs recorded from interneurons (**c**) and summary data (**d**) of the effect on mIPSCs frequency and amplitude of interneurons before (in black), during (in green) and after (in blue) NG2 glia photostimulation. The frequency of mIPSCs of interneurons is significantly enhanced after 15 Hz, 90 sec photostimulation of NG2 glia. The intensity of illumination is 5 mW/mm^2. n = 11 cells from 4 mice at postnatal 4-6 weeks, Tukey-Kramer Multiple Comparisons

Test. * indicates $P < 0.05$, n.s. indicates not significant. Data are presented as mean values ± SEM and error bar represents SEM.

Supplementary Figure 4

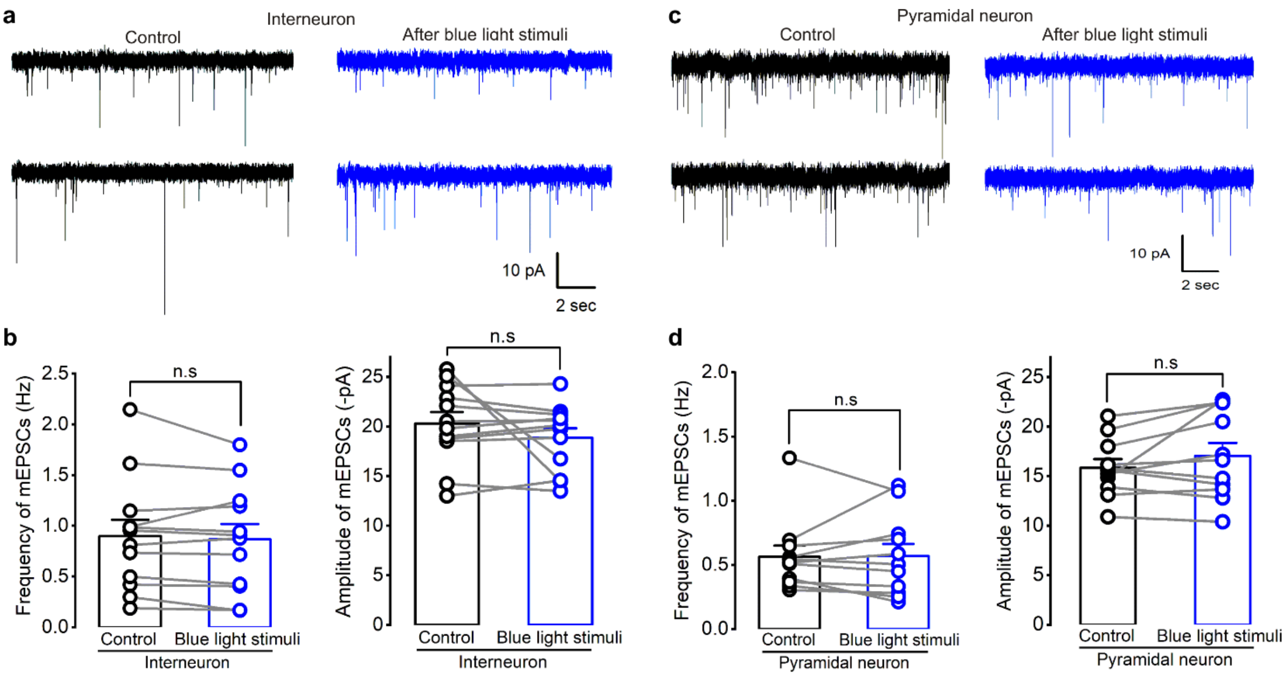

Supplementary Fig. 4 Representative traces of miniature excitatory postsynaptic currents (mEPSCs) from interneurons (**a**) and summary data (**b**) of the effect of NG2 glia photostimulations on mEPSCs frequency and amplitude. The average frequency of mEPSCs: 0.90 ± 0.16 Hz pre- vs. 0.86 ± 0.15 Hz post-NG2 glia photoactivation; the average amplitude of mEPSCs: -20.31 ± 1.15 pA pre- vs. -18.87 ± 0.96 pA post-NG2 glia photoactivation, respectively, n = 12 cells from 3 mice, two-tailed paired t-test, n.s. indicates not significant. **c-d**, Representative traces of mEPSCs (**c**) from pyramidal neurons and summary data (**d**) of the effect of NG2 glia photostimulations on mEPSCs frequency and amplitude. The average frequency of mEPSCs: 0.56 ± 0.09 Hz pre- vs. 0.57 ± 0.10 Hz post-NG2 glia photoactivation; the average amplitude of mEPSCs: -15.84 ± 0.87 pA pre- vs. -17.06 ± 1.31 pA post-NG2 glia photoactivation, respectively, n = 11 cells from 3 mice, two-tailed paired t-test, n.s. indicates not significant. Data are presented as mean values ± SEM and error bar represents SEM.

Supplementary Figure 5

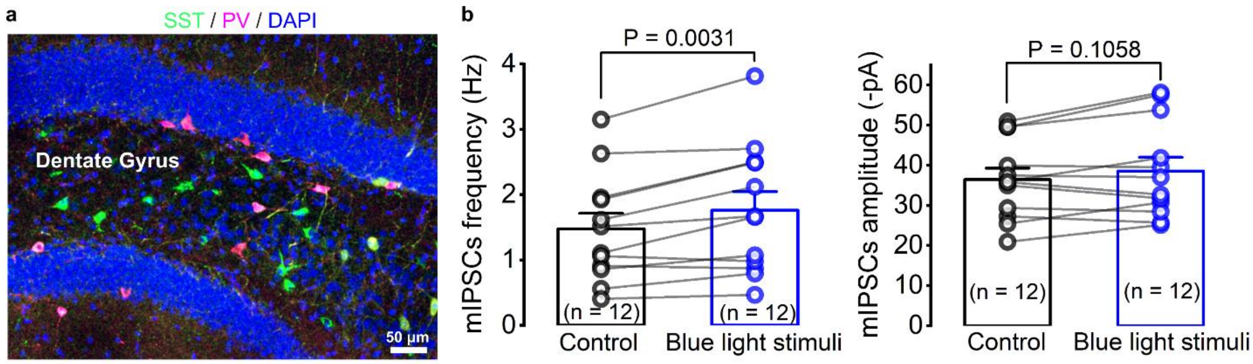

Supplementary Fig. 5 The effect of NG2 glia photoactivation on mIPSCs of interneurons in the hilus of hippocampal dentate gyrus at postnatal 4-6 weeks. **a**, The representative image shows that most PV positive- (in magenta) and SST positive- (in green) interneurons are located in the hilus region of hippocampal dentate gyrus. Scale bar, 50 µm. **b**, Summary bar graphs show that a significant increase of mIPSCs frequency of interneurons in the hilus occurs after NG2 glia photostimulations. The average frequency of mIPSCs: 1.48 ± 0.24 Hz for control vs. 1.76 ± 0.29 Hz after NG2 glia photoactivation, n = 12 interneurons recorded from 4 mice. P = 0.0031, two-tailed paired t-test. The average amplitude of mIPSCs: -36.45 ± 2.84 pA for control vs. -38.53 ± 3.46 pA after NG2 glia photoactivation, n = 12 interneurons from 4 mice. P = 0.1058, two-tailed paired t-test. Data are presented as mean values ± SEM and error bar represents SEM.

Supplementary Figure 6

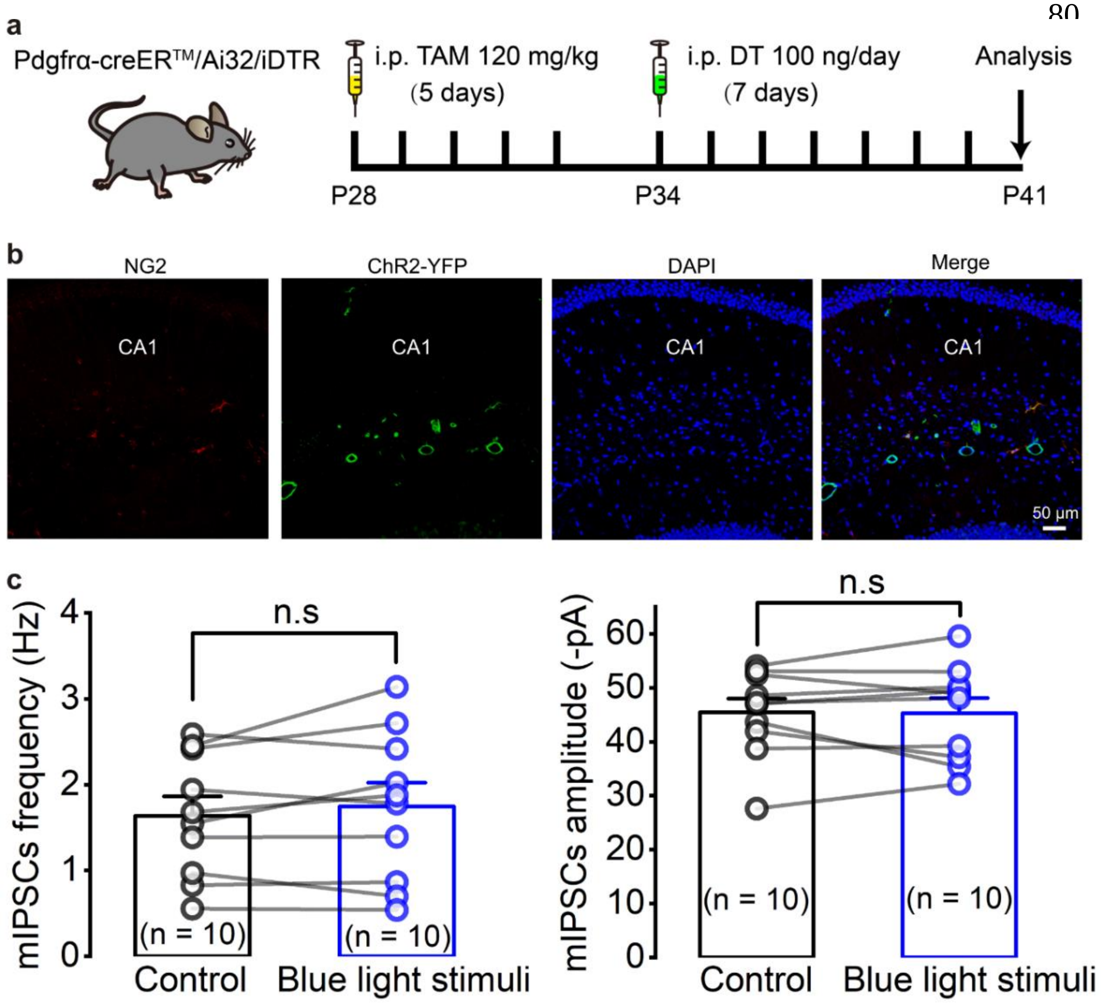

Supplementary Fig. 6 The increased frequency of mIPSCs of interneurons is abolished after NG2 glia ablation. **a**, Paradigm used for NG2 glial cell ablation in the Pdgfrα-creER^{TM}/ChR2-eYFP/iDTR mouse at postnatal 4-6 weeks using systemic Diphtheria Toxin (DT) administration. DT (100 ng) was injected once daily for 7 days. **b**, Representative images show NG2 glia ablation in the hippocampus of Pdgfrα-creER^{TM}/ChR2-eYFP/iDTR mouse. Scale bar, 50 µm. **c**, Summary bar graphs show that the effect on mIPSCs frequency of interneurons is abolished when NG2 glia photostimulations after cells' ablation. n = 10 cells from 3 mice at postnatal 4-6 weeks. The average frequency of mIPSCs: 1.64 ± 0.23 Hz pre- vs. 1.74 ± 0.28 Hz post-NG2 glia photoactivation, P = 0.2938, two-tailed paired t-test; the average amplitude of mIPSCs: -45.43 ± 2.53 pA pre- vs. -45.30 ± 2.79 pA post-NG2 glia photoactivation, P = 0.9228, two-tailed paired t-test, n.s.

indicates not significant. Data are presented as mean values ± SEM and error bar represents SEM.

Supplementary Figure 7

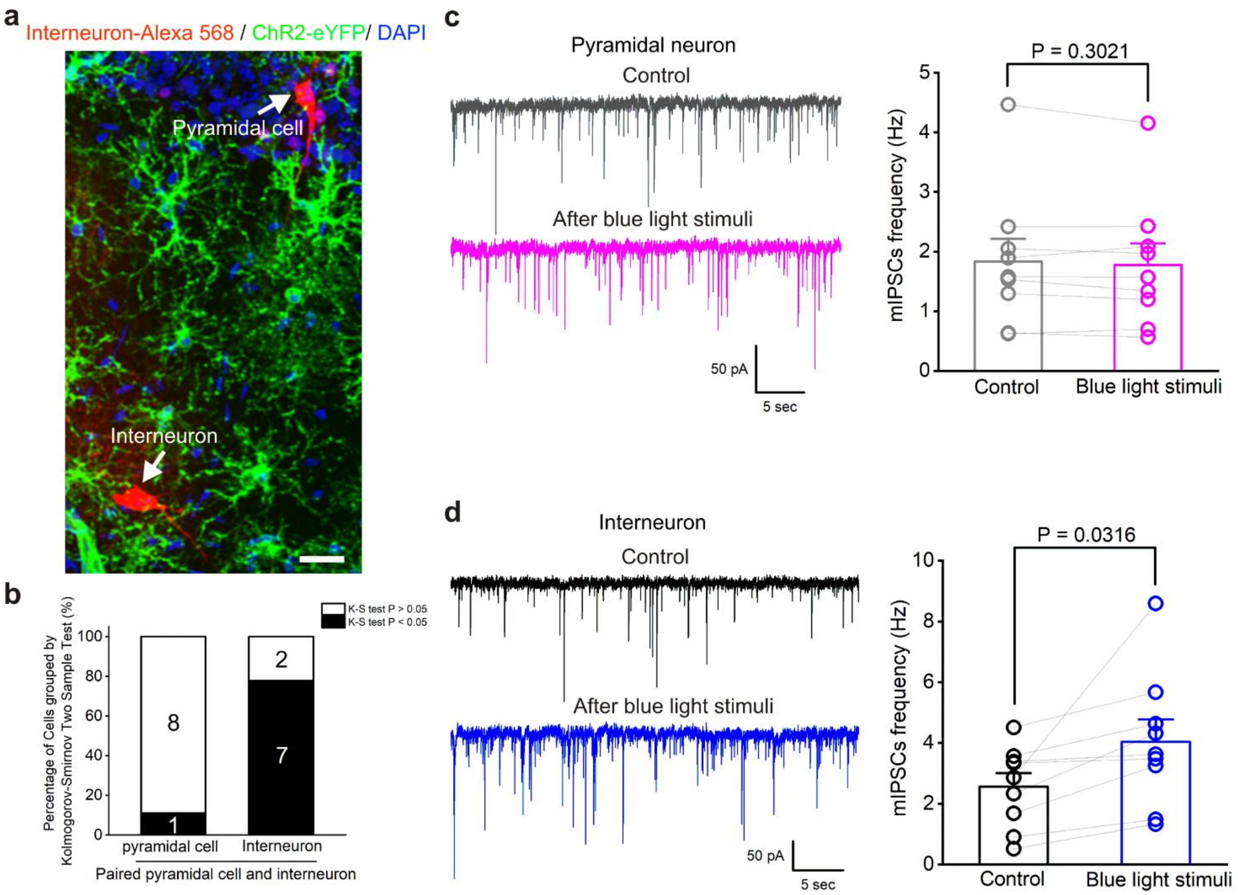

Supplementary Fig. 7 The effect of NG2 glia photoactivation on mIPSCs of dual patched pyramidal cells and interneurons in hippocampal CA1 region. **a**, The representative image shows the distinct morphology of a dual-patched interneuron and pyramidal cell loaded with Alexa Fluor 568 in the patch pipettes in hippocampal CA1 region. The red fluorescence dialyzed neurons are indicated by the arrows. The green fluorescence labeled cells indicate the ChR2-expressed NG2 glia. Scale bar, 20 µm. **b**, Summary bar graph shows the percentage of cells for the increase of mIPSCs frequency from 9 pairs of recorded pyramidal cells and interneurons after NG2 glia photoactivation by using Kolmogorov-Smirnov Two Sample Test analysis. Note that the recorded interneurons are typically chosen where the proximity to its closest NG2 glia soma is less than 30 µm. The pyramidal neurons located in CA1 pyramidal cell layer are chosen for recording. **c-d**, Representative mIPSCs traces recorded from a pair recording of CA1 pyramidal cell (**c**) and interneuron (**d**) before and after NG2 glia photostimulation (15 Hz, 90 sec). Summary bar graphs show a significant increase in frequency of mIPSCs onto

interneurons but no effect on pyramidal neurons. n = 9 pairs for each group, Data are presented as mean values ± SEM and error bar represents SEM.

Supplementary Figure 8

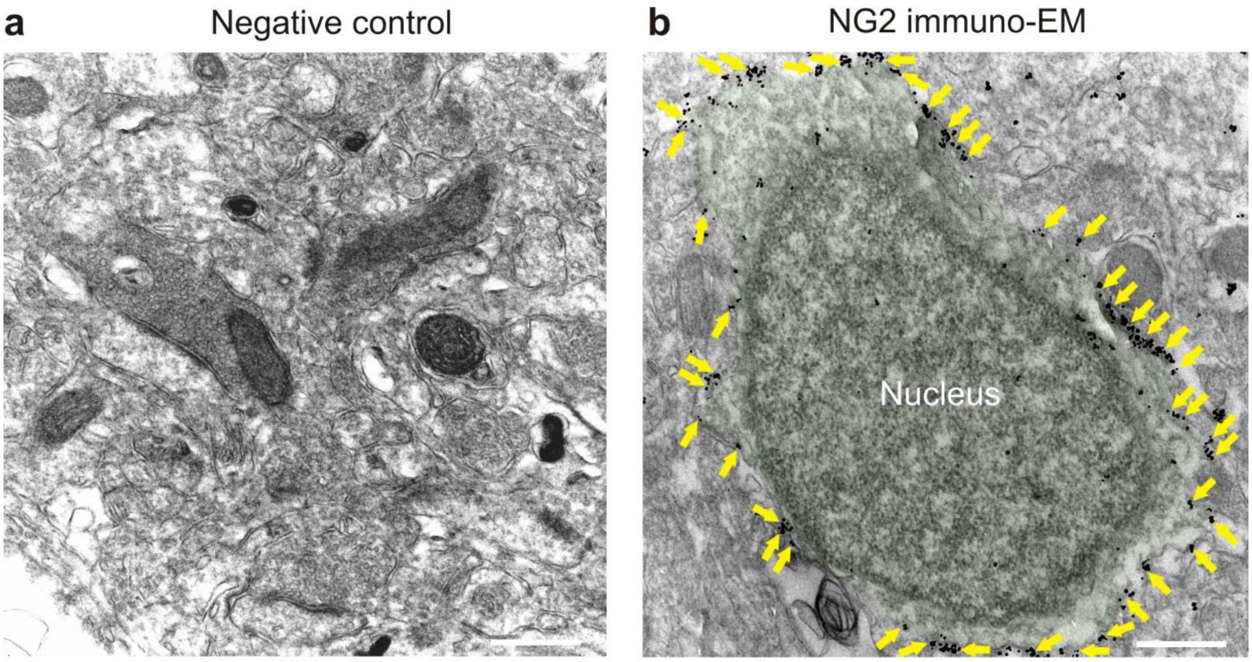

Supplementary Fig. 8 The specificity of NG2 immuno-electron microscopy in adult hippocampal tissues. **a**, Representative image for the negative control shows no staining of NG2 immunogold particles when the primary antibody is omitted in the hippocampal CA1 region from C57BL/6 wild type mice at postnatal 6-8 weeks. **b**, Representative image shows apparent immunogold particles that label for the anti-NG2 antibody are contiguously distributed along the plasma membrane, with a few immunogold particles scattered in the cytoplasm of NG2 glia (in green) in C57BL/6 wild type mouse hippocampus at postnatal 6-8 weeks. The yellow arrows indicate the NG2-immunogold particles. Scale bars, 500 nm.

Supplementary Figure 9

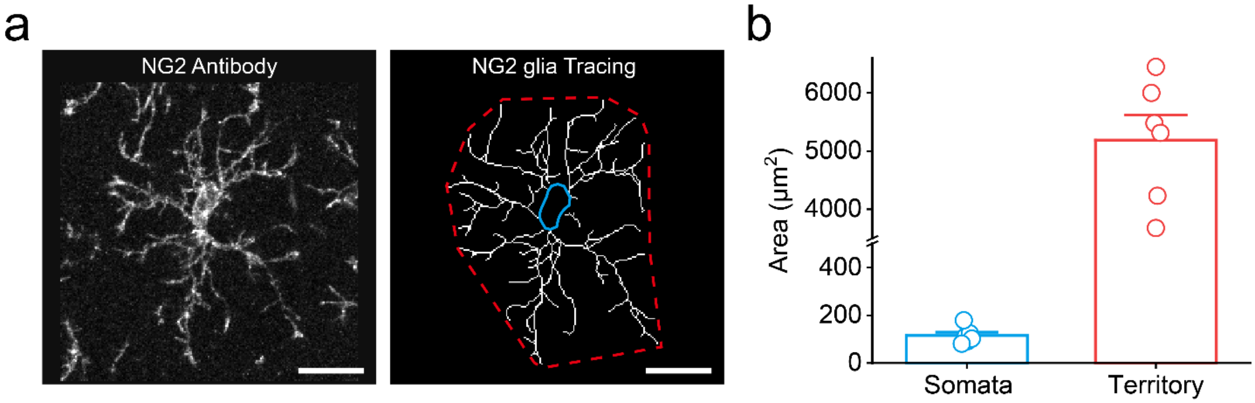

Supplementary Fig. 9 The territory of NG2 glia in CA1 region of adult mouse hippocampus. **a**, Representative images show the morphology of one NG2 antibody-labeled glial cell (left panel) in adult hippocampal CA1 region and its 8-bit constructed image (right panel) by using the Fiji plugin Simple Neurite Tracer. The soma and its territory boundary of each NG2 glial cell are outlined in blue and red, respectively. Scale bars, 20 μm. **b**, The bar graph shows the average area of the corresponding soma (115.2 ± 14.2 μm^2, n = 6 cells) and the territory of NG2 glia (5191.3 ± 429.6 μm^2, n = 6 cells). Data are presented as mean values ± SEM and error bar represents SEM.

Supplementary Figure 10

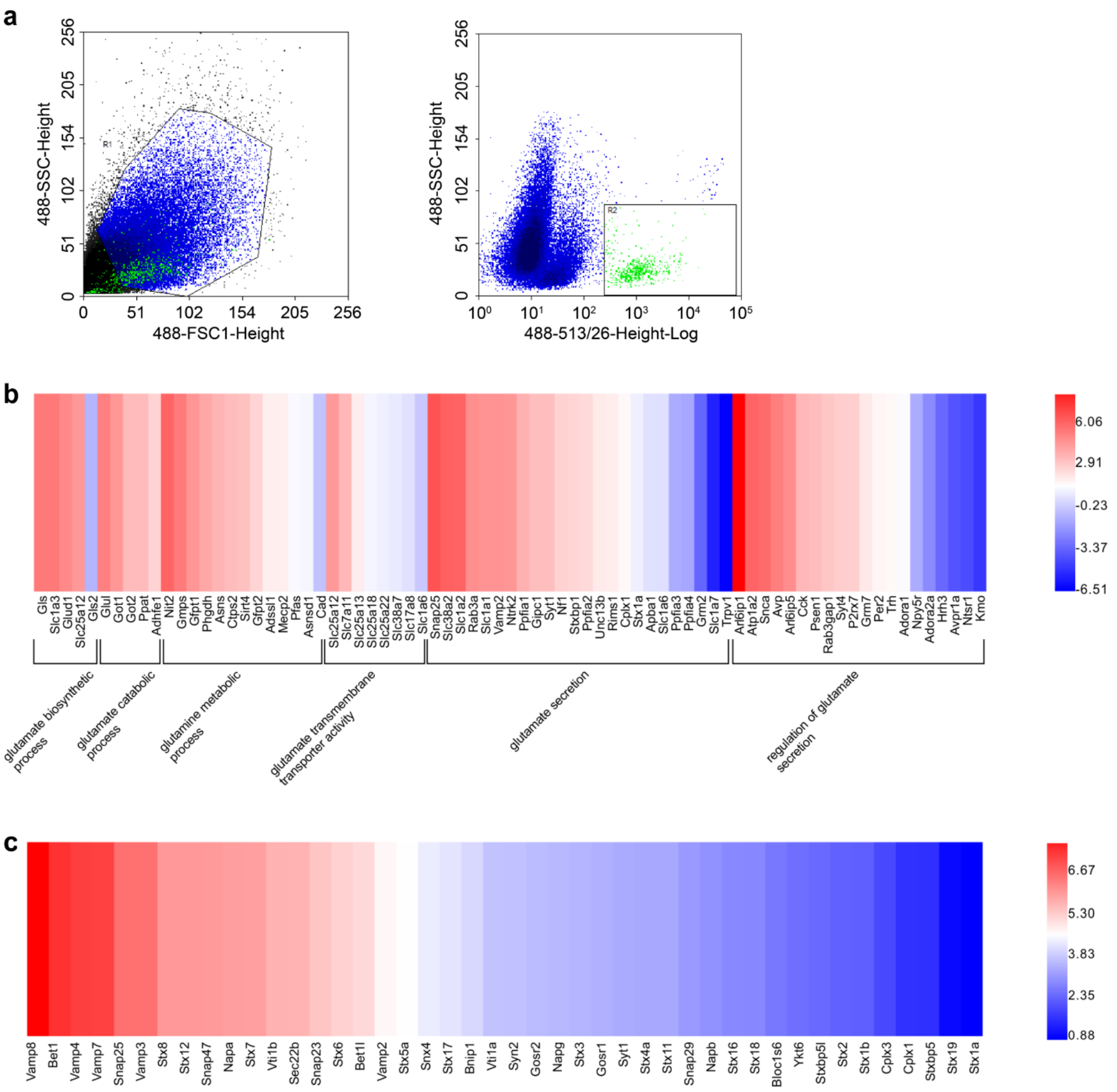

Supplementary Fig. 10 Bulk-RNA sequencing analysis in sorted NG2 glia by FACS in Pdgfrα-creER^{TM};mGFP hippocampus. **a,** NG2 glial cells harvested from postnatal 3-4 week old Pdgfrα-creER^{TM};mGFP mice before (blue) and after (green) sorted by FACS. Green fluorescently labeled cells are purified NG2+ cells for sequential experiments. **b-c,** The transcriptomic data analysis reveals the genes related with glutamate biosynthetic processes, catabolic, metabolic, secretion and transmembrane transporter activity (**b**) and a soluble N-ethylmaleimide-sensitive fusion protein attachment protein receptor (SNARE) complex (**c**) from bulk RNA-

sequencing profiling of NG2 glia isolated from Pdgfrα-creER^{TM};mGFP mice. The Log2(FPKM) expression levels were averaged from 4 mice at postnatal 3-4 weeks.

Supplementary Figure 11

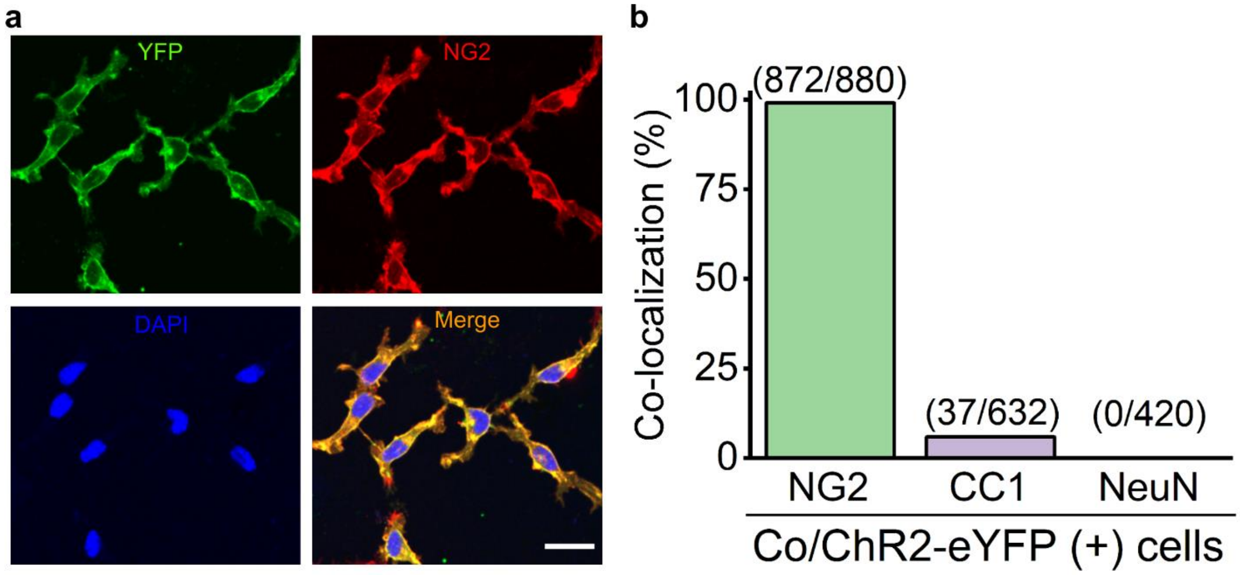

Supplementary Fig. 11 NG2 glia expression in purified OPCs obtained from Pdgfrα-creER^{TM};ChR2-eYFP newborn mice. **a**, Representative images of immunocytochemistry showing a 98.77 ± 0.46% (n = 880 cells from 4 mice) colocalization between YFP-labeled cells and NG2 antibody. Scale bar, 20 µm. **b**, Bar graph summary showing the colocalization rate between YFP-labeled cells and OPC marker NG2, mature oligodendrocyte marker CC1 and neuronal marker NeuN in purified OPCs.

Supplementary Figure 12

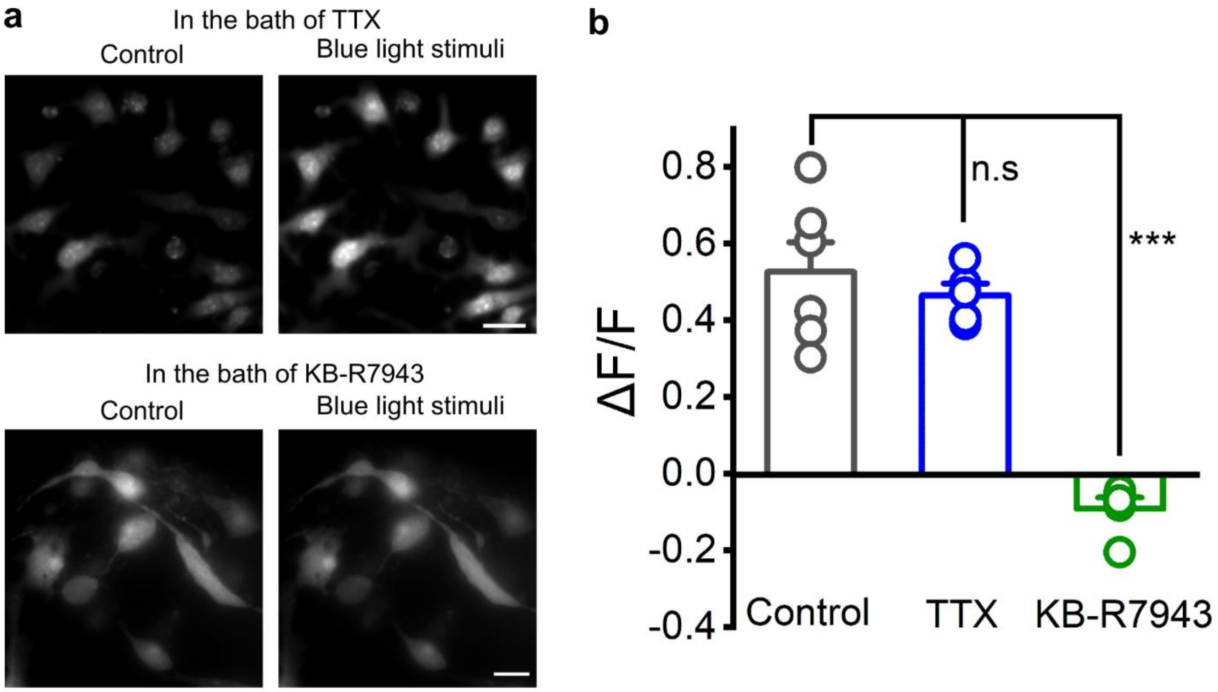

Supplementary Fig. 12 The effect on $[Ca^{2+}]_i$ in purified NG2 cells by sodium channel blocker and Na^+-Ca^{2+} exchangers inhibitor. **a**, Representative Ca^{2+} images showing that blue light stimulation-induced $[Ca^{2+}]_i$ elevations in purified NG2 glial cells from Pdgfrα-creER^{TM};ChR2-eYFP mice are not abolished by sodium channel blocker TTX (1 µM) but are blocked by Na^+-Ca^{2+} exchangers inhibitor KB-R7943 (100 µM). **b**, Bar graph shows the change of $[Ca^{2+}]_i$ intensity in ChR2-expressing NG2 cells which are loaded with Rhod2-AM at 5 µM after blue light stimulation (15 Hz, 60 s). The average peak increase of $[Ca^{2+}]_i$ intensity: Control, 0.53 ± 0.08, n = 53 cells from 6 FOV (field of view); in TTX, 0.47 ± 0.03, n = 50 cells from 5 FOV; in KB-R7943, -0.09 ± 0.03. n = 53 cells from 5 FOV. $P > 0.05$, control vs. TTX group; $P < 0.001$, control vs. KB-R7943 group, one-way ANOVA Tukey-Kramer Multiple Comparisons test. Data are averaged from 3 mice in three independent experiments. Data are presented as mean values ± SEM and error bar represents SEM.

Supplementary Figure 13

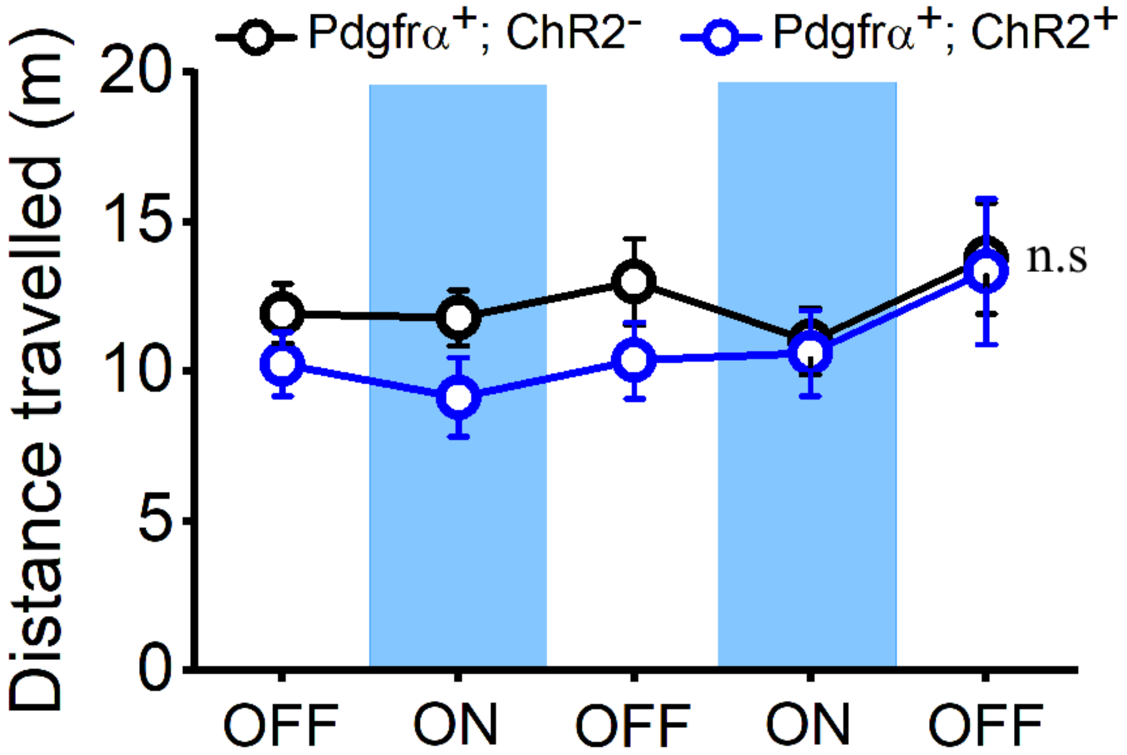

Supplementary Fig. 13 Bar graph shows the average of total distance travelled by Pdgfrα-creER^{+};ChR2^{+} mice and the control mice over 25 min, including blue light stimulations (20 Hz, 20 msec, 10 s ON / OFF) in an open field chamber, divided into 5-min epochs for the two experimental groups. Distance travelled: 12.08 ± 1.06 m vs. 10.25 ± 1.07 m in 0-5 min OFF, P = 0.2358; 11.71 ± 1.00 m vs. 9.14 ± 1.33 m in 5-10 min ON, P = 0.1348; 13.14 ± 1.56 m vs. 10.36 ± 1.28 m in 10-15 min OFF, P = 0.1810; 10.66 ± 1.14 m vs. 10.60 ± 1.43 m in 15-20 min ON, P = 0.9742; 13.54 ± 1.99 m vs. 13.33 ± 2.44 m in 20-25 min OFF, P = 0.9323. n = 12 Pdgfrα-creER^{+};ChR2^{-} and Pdgfrα-creER^{+};ChR2^{+} mice at postnatal 8-12 weeks for each group. Two-tailed unpaired t-test (0-5, 5-10, 10-15, 15-20 min) and two-tailed Mann-Whitney test (20-25 min). n.s. indicates not significant. Data are presented as mean values ± SEM and error bar represents SEM.

Supplementary Figure 14

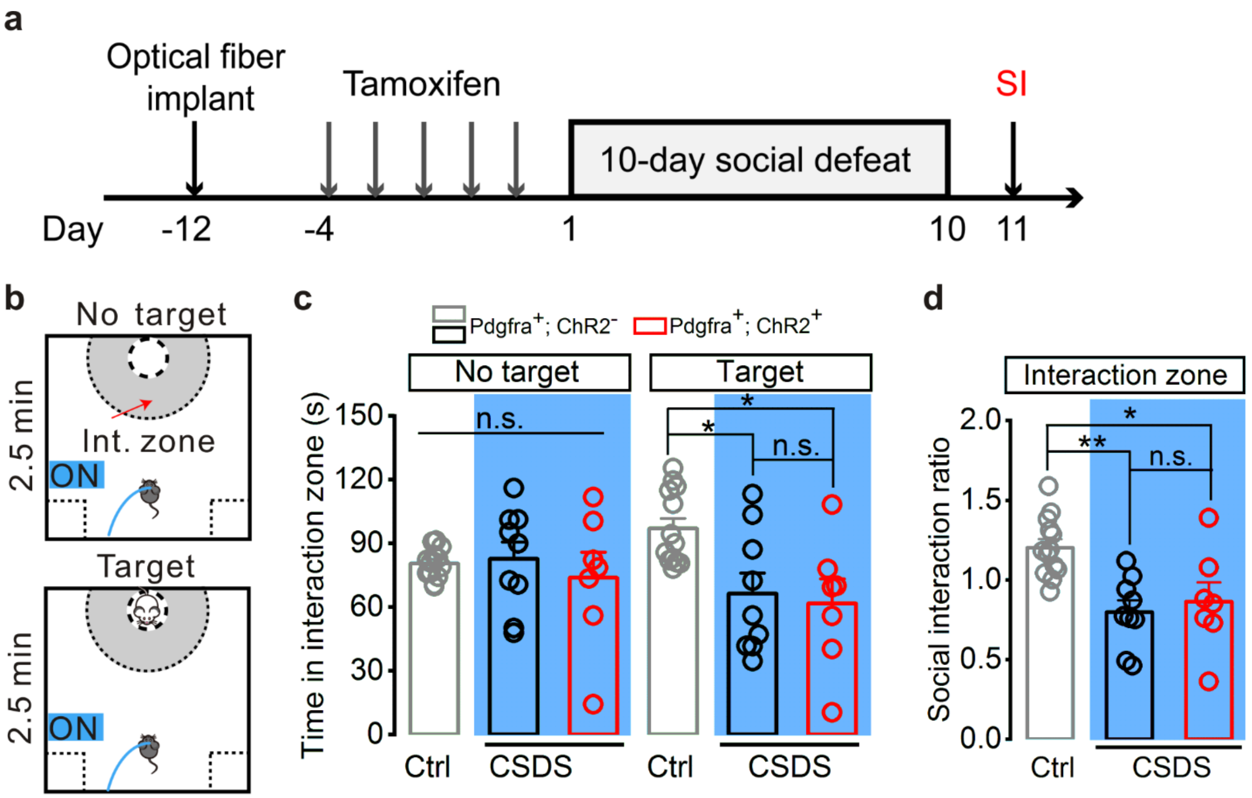

Supplementary Fig. 14 NG2 glia-photoactivation in adult hippocampus does not alter social avoidance behavior in chronic social defeat stress (CSDS) mice. **a**, Schematic illustrating the experimental approach for social interaction (SI) test. After 10 consecutive days of CSDS, experimental animals were housed singly and tested 24 h later for SI test. **b**, Cartoon illustrating the procedure for intermittent photoactivation (blue light, 20 Hz, 20 msec, 10 s ON / OFF) of ChR2-expressing NG2 glia in adult hippocampus during 2.5 min × 2 SI test. Non-CSDS Pdgfrα^{+};ChR2^{-} mice and CSDS Pdgfrα^{+};ChR2^{-} mice as its control. **c,** Quantification of time in the interaction zone: No target: Pdgfra^{+};ChR2^{-}-Ctrl (80.58 ± 1.96 sec) vs. Pdgfra^{+};ChR2^{-}-CSDS (82.81 ± 7.93 sec) vs. Pdgfra^{+};ChR2^{+}-CSDS (73.88 ± 12.05 sec), $P > 0.05$, Kruskal-Wallis test. Target: Pdgfra^{+};ChR2^{-}-Ctrl (96.97 ± 4.83 sec) vs. Pdgfra^{+};ChR2^{-}-CSDS (66.41 ± 9.68 sec), $P < 0.05$, one-way ANOVA test; Pdgfra^{+};ChR2^{-}-Ctrl (96.97 ± 4.83 sec) vs. Pdgfra^{+};ChR2^{+}-CSDS (61.75 ± 11.62 sec), $P < 0.05$, one-way ANOVA test. * indicates $P < 0.05$. n.s. indicates not significant. n = 13, 9, 7 mice at postnatal 8-12 weeks for each group, respectively. **d,** SI ratio in the interaction zone: Pdgfra^{+};ChR2^{-}-Ctrl (1.20 ±

0.05) vs. Pdgfra^{+};ChR2^{-}-CSDS (0.80 ± 0.07), P ＜ 0.01, one-way ANOVA test. Pdgfra^{+};ChR2^{-}-Ctrl (1.20 ± 0.05) vs. Pdgfra^{+};ChR2^{+}-CSDS (0.86 ± 0.12), P ＜ 0.05, one-way ANOVA test. * indicates P < 0.05, ** indicates P < 0.01, n.s. indicates not significant. n = 13, 9, 7 mice at postnatal 8-12 weeks for each group, respectively. Data are presented as mean values ± SEM and error bar represents SEM.

Supplementary Figure 15

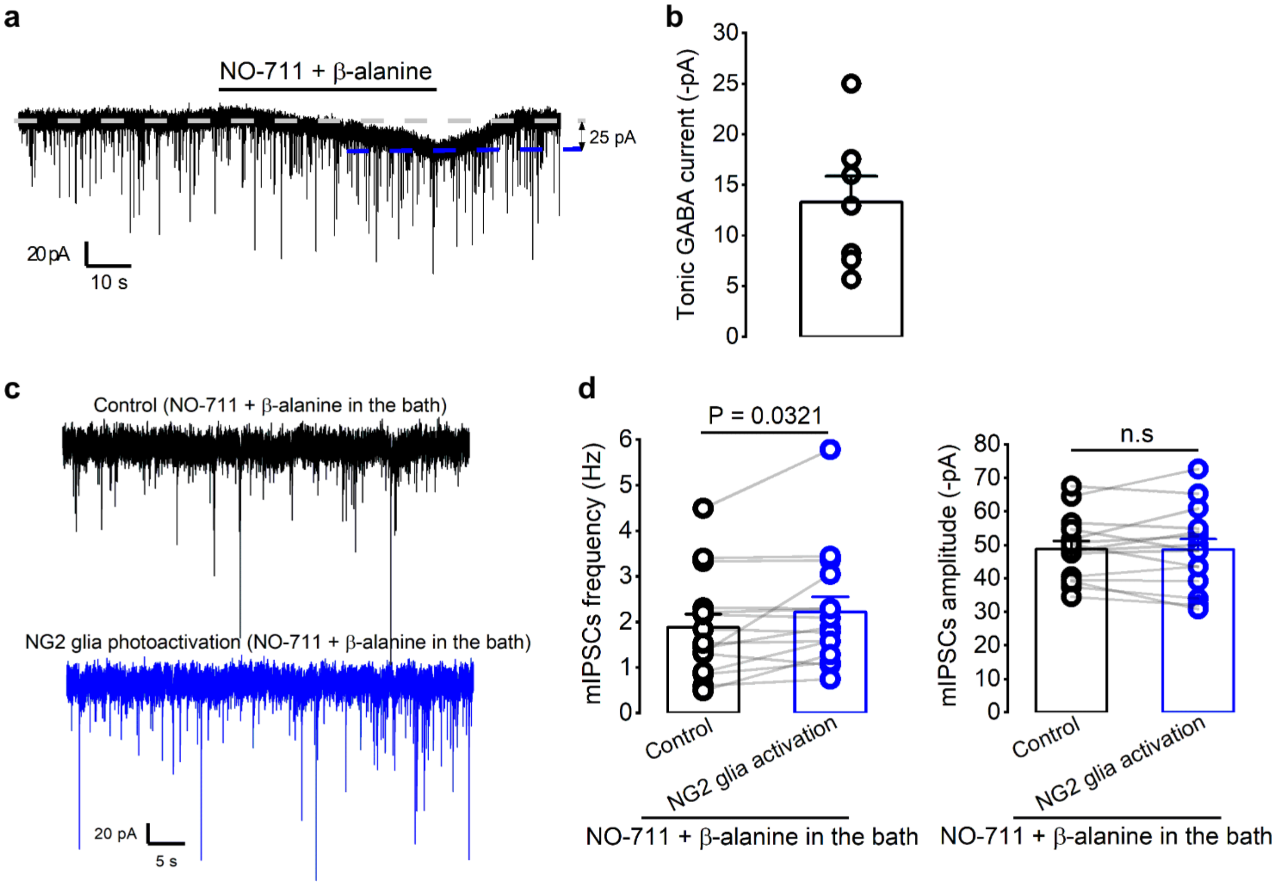

Supplementary Fig. 15 Enhancement of mIPSCs frequency onto interneurons after NG2 glia photoactivation is not abolished by GABA transport regulation. **a**, Representative trace showing that an inward GABA current is induced in a whole-cell patched interneuron after bath-applied GABA transporter blockers including NO-711 (10 µM) and β-alanine (100 µM) to block GAT-1 and GAT-3 which are known to widely express in the hippocampus. **b**, Summary graph shows an average of -13.28 ± 2.58 pA tonic GABA current in 7 recorded interneurons induced by the GABA transporter antagonists. **c-d**, Representative traces (**c**) and bar graphs (**d**) showing an increased frequency of mIPSCs onto an interneuron after NG2 glia photoactivation with GABA transporter blockers NO-711 and β-alanine in the bath. Average frequency of mIPSCs: 1.88 ± 0.29 Hz pre- vs. 2.22 ± 0.33 Hz post-NG2 glia photoactivation, respectively. n = 15 cells from 4 Pdgfrα-creER$^+$;ChR2$^+$ mice at postnatal 4-6 weeks, two-tailed paired t-test, P = 0.0321. Average amplitude of mIPSCs: -48.74 ± 2.48 pA pre- vs. -48.68 ± 3.12 pA post-NG2 glia photoactivation, respectively. n = 15 cells from 4 Pdgfrα-creER$^+$;ChR2$^+$ mice at

postnatal 4-6 weeks, two-tailed paired t-test, n.s. indicates not significant. Data are presented as mean values ± SEM and error bar represents SEM.

Supplementary Table 1. List of primers used for single-cell RT PCR.

Name	Forward	Reverse	Product size (bp)
Gad1	CATGGCTGCTCGTTACAAGTA	AACAGTCGTGCCTGCGGTTGC	268
Gad2	GCACTCACCAGGAAAGGAAC	CAGCAGTGCCCAGGCTCATCG	199
Pdgfrα	ACAGAGACTGAGCGCTGACA	CTCGATGGTCTCGTCCTCTC	178
Egfp	CACATGAAGCAGCACGACTT	AGTTCACCTTGATGCCGTTC	264
Gapdh	TGGACTGTGGTCATGAGCCC	GGCAAATTCAACGGCACAGT	317
