# Supplementary material for: NG2 glia-derived GABA release tunes inhibitory synapses and contributes to stress-induced anxiety
Source: Nat Commun. 2021 Sep 30;12:5740. doi: 10.1038/s41467-021-25956-y (PMC8484468; doi:10.1038/s41467-021-25956-y)
Supplement: Supplementary file 2 — Reporting Summary [file 41467_2021_25956_MOESM2_ESM.pdf]

nature portfolio

Corresponding author(s): Xiaoping Tong

Last updated by author(s): Aug 25, 2021

Reporting Summary

Nature Portfolio wishes to improve the reproducibility of the work that we publish. This form provides structure for consistency and transparency in reporting. For further information on Nature Portfolio policies, see our Editorial Policies and the Editorial Policy Checklist.

Statistics

For all statistical analyses, confirm that the following items are present in the figure legend, table legend, main text, or Methods section.

n/a	Confirmed	
☐	☒	The exact sample size (n) for each experimental group/condition, given as a discrete number and unit of measurement
☐	☒	A statement on whether measurements were taken from distinct samples or whether the same sample was measured repeatedly
☐	☒	The statistical test(s) used AND whether they are one- or two-sided<br>*Only common tests should be described solely by name; describe more complex techniques in the Methods section.*
☒	☐	A description of all covariates tested
☐	☒	A description of any assumptions or corrections, such as tests of normality and adjustment for multiple comparisons
☐	☒	A full description of the statistical parameters including central tendency (e.g. means) or other basic estimates (e.g. regression coefficient) AND variation (e.g. standard deviation) or associated estimates of uncertainty (e.g. confidence intervals)
☐	☒	For null hypothesis testing, the test statistic (e.g. F, t, r) with confidence intervals, effect sizes, degrees of freedom and P value noted<br>*Give P values as exact values whenever suitable.*
☒	☐	For Bayesian analysis, information on the choice of priors and Markov chain Monte Carlo settings
☒	☐	For hierarchical and complex designs, identification of the appropriate level for tests and full reporting of outcomes
☐	☒	Estimates of effect sizes (e.g. Cohen's d, Pearson's r), indicating how they were calculated

Our web collection on statistics for biologists contains articles on many of the points above.

Software and code

Policy information about availability of computer code

Data collection	Electrophysiological data were acquired with a MultiClamp 700B amplifier (Molecular Devices, Sunnyvale, CA, USA), sampled at 20 kHz using Digidata 1550A (Molecular Devices).<br>IHC images were acquired on a Leica TCS SP8 confocal microscope with Leica LAS X Core (3.4.2.18368) software.<br>Immuno-electron images were acquired on Tecnai G2 Spirit 120kV transmission electron microscopy (FEI).<br>Flow cytometry data were collected on Beckman Coulter Moflo Astrios cell sorter.<br>TIRFM imaging was collected on Olympus IX-83 inverted microscope equipped with a UAPON 100 × OTIRF NA 1.49 OIL objective.<br>Calcium imaging was collected on Fluoview FVMPE-RS two-photon microscope with a 25 × 1.05 N.A. water-immersion objective.<br>HPLC (high performance liquid chromatography) analysis was performed using the Agilent 1260 series neurotransmitter analyzer.<br>For RNA-sequencing, FPKM values of each gene were obtained by Cufflinks 2.2.1 using genome annotation from UCSC.
Data analysis	Electrophysiological data were analyzed with Clampfit 10.5.<br>Image data were analyzed by ImageJ 1.52a (US National Institutes of Health).<br>The graphs were created in Origin 8 and assembled in CorelDraw 12. No unpublished custom algorithms were used.<br>All statistical tests were run in GraphPad InStat 3.

For manuscripts utilizing custom algorithms or software that are central to the research but not yet described in published literature, software must be made available to editors and reviewers. We strongly encourage code deposition in a community repository (e.g. GitHub). See the Nature Portfolio guidelines for submitting code & software for further information.

Data

Policy information about availability of data

All manuscripts must include a data availability statement. This statement should provide the following information, where applicable:

- Accession codes, unique identifiers, or web links for publicly available datasets
- A description of any restrictions on data availability
- For clinical datasets or third party data, please ensure that the statement adheres to our policy

All data needed to evaluate the conclusions of the study are present in the paper or the supplementary materials. The Raw data for each figure has been provided as a Source Data file. The accession number for the bulk and single-cell RNA-seq data is SRP215327 in SRA and GSE162049 in GEO, respectively.

Field-specific reporting

Please select the one below that is the best fit for your research. If you are not sure, read the appropriate sections before making your selection.

[x] Life sciences [ ] Behavioural & social sciences [ ] Ecological, evolutionary & environmental sciences

For a reference copy of the document with all sections, see nature.com/documents/nr-reporting-summary-flat.pdf

Life sciences study design

All studies must disclose on these points even when the disclosure is negative.

Sample size	No statistical methods were used to pre-determine sample size. The sample sizes of each set of animals were determined according to previous studies performed by our group and other scholars and were fixed in a prospective manner. The exact number of animal used in individual experiments are indicated in the figure legends.
Data exclusions	For electrophysiological experiments, membrane access resistence of whole-cell patched recording was monitored before and after recording and the data which with a >20% change was excluded from the analysis. For behavioral tests. animals falling from the apparatus during the test, software failure during behavioral data collection or incorrect placement of the optical fiber were removed from the analysis.
Replication	All experiments were independently replicated at least three times except single-cell RNA sequencing. Replicated experiments yielded the same results.
Randomization	All samples used in the study were randomly allocated into different experimental groups.
Blinding	Blinding was not relevant for this study, as the specific cell type and animals need to be identified clearly by the fluorescent labeling and/or genotyping. However, personnel who performed the fluorescence-activated cell sorting (FACS), RNA-sequencing and immuno-electron microscopy experiments did not know the samples we collected and/or analysis.

Reporting for specific materials, systems and methods

We require information from authors about some types of materials, experimental systems and methods used in many studies. Here, indicate whether each material, system or method listed is relevant to your study. If you are not sure if a list item applies to your research, read the appropriate section before selecting a response.

Materials & experimental systems

n/a	Involved in the study	
[ ]	[x]	Antibodies
[ ]	[x]	Eukaryotic cell lines
[x]	[ ]	Palaeontology and archaeology
[ ]	[x]	Animals and other organisms
[x]	[ ]	Human research participants
[x]	[ ]	Clinical data
[x]	[ ]	Dual use research of concern

Methods

n/a	Involved in the study	
[x]	[ ]	ChIP-seq
[ ]	[x]	Flow cytometry
[x]	[ ]	MRI-based neuroimaging

Antibodies

Antibodies used	The primary antibodies used include: rabbit antibody to NG2 (Millipore, AB5320), goat antibody to Pdgfrα (R&D Systems, AF1062), mouse antibody to Olig2 (Millipore, MABN50), mouse antibody to CC1 (Millipore, OP80), rabbit antibody to GFAP (Abcam, ab7260), chicken antibody to GFP (Abcam, ab13970), mouse antibody to cFos (Abcam, ab208942), mouse antibody to NeuN (Abcam, ab104224), rabbit antibody to CCK-8 (Sigma, C2581), rabbit antibody to NPY (Cell Signaling, 11976S), mouse antibody to PV (Sigma, P3088), rat antibody to SST (Millipore, MAB354), guinea pig antibody to vGluT2 (Synaptic Systems, 135404), rabbit antibody to

	VAMP-2 (Alomone labs, ANR-007), mouse antibody to Gephyrin (Synaptic Systems, 147011), rabbit antibody to CaMKII (Abcam, ab52476), mouse antibody to GAD67 (Millipore, MAB5406) and rabbit antibody to GABA (Sigma, A2052). The corresponding secondary antibodies include donkey anti-rabbit Alexa Fluor 568 (Invitrogen, A10042), donkey anti-mouse Alexa Fluor 647 (Invitrogen, A31571), goat anti-chicken Alexa Fluor 488 (Invitrogen, A11039), donkey anti-goat Alexa Fluor 488 (Invitrogen, A11055), goat anti-rat Alexa Fluor 647 (Invitrogen, A21247), goat anti-rabbit Alexa Fluor 647 (Cell Signaling, 4414S), goat anti-rabbit Alexa Fluor 488 (Cell Signaling, 4412S), goat anti-guinea pig Alexa Fluor 647 (Invitrogen, A21450), rabbit Anti-mouse IgG antibody, HRP conjugate (Millipore, AP160P) and gold-conjugated anti-rabbit IgG (Jackson ImmunoReseach, 111-205-144).
Validation	All antibodies have been validated in the literature and/or in Antibodypedia for use in mouse immunohistochemistry and western blot. To further validate the antibodies on our hands, we confirmed that each antibody stained in the expected cellular patterns and brain-wide distributions for immunohistochemistry.

Eukaryotic cell lines

Policy information about cell lines

Cell line source(s)	HEK293T cells (human, ATCC, CRL-3216™), MOLT-4 T lymphoblast (human, ATCC, CRL-1582™).
Authentication	None of the cell lines have been authenticated.
Mycoplasma contamination	Cell lines were not tested for mycoplasma contamination but no indication of contamination was observed.
Commonly misidentified lines (See ICLAC register)	No commonly misidentified cell lines were used.

Animals and other organisms

Policy information about studies involving animals; ARRIVE guidelines recommended for reporting animal research

Laboratory animals	All mouse experiments were approved by the Animal Ethics Committee of Shanghai Jiao Tong University School of Medicine (AAALAC accreditation Unit, 001670) and the Institutional Animal Care and Use Committee, the protocol number is A-2018-028. All mice were kept on a C57BL/6 background and under a standard conditions with temperatures of 21–23°C, 40–60% humidity, and a 12 hr -12 hr light-dark cycle with food and water provided ad libitum from the cage lid. Pdgfrα-creER™(JAX strain 018280, B6N.Cg-Tg(Pdgfra-cre/ERT)467Dbe/J), ChR2(H134R)-eYFP (Ai32) (JAX strain 024109, B6.Cg Gt(ROSA)26Sortm32-(CAG-COP4*H134R/EYFP)Hze/J), Rosa26-mGFP (JAX strain 007676, mT/mG B6.129(Cg)-Gt(ROSA)26Sortm4(ACTB-tdTomato,-EGFP)Luo/J) mice were obtained from the Jackson Laboratory (U.S.A.). GCaMP6sflox (JAX strain 024106, 129S6-Gt(ROSA)26S or Ai96) mice were a gift from Prof. Nanjie Xu, ROSA26iDTR (JAX strain 007900, Gt(ROSA)26Sortm1(HBEGF)Awai/J) mice were a gift from Prof. Qian Li, GAD67-GFP knock-in mice (RBRC03674, ICR.Cg-Gad1<tm1.1Tama>) were a gift from Prof. Jiangteng Lv at Shanghai Jiao Tong University School of Medicine (Shanghai, China). NG2-creER™ (JAX strain 008538, B6.Cg-Tg(Cspg4-cre/Esr1*)BAkik/J) was gifted from Prof. Chong Liu at Zhejiang University (Zhejiang, China). C57BL/6J mice (JAX strain 000664) were obtained from the Slac Laboratory Animal (Shanghai, China). As for the NG2 glia culture, mouse cortices including hippocampus were isolated from Pdgfrα-creER™; ChR2-eYFP pups at postnatal day 1. We used the mice at postnatal 3-6 weeks to test Electrophysiological recordings, Immunohistochemistry, FACS, Single-cell RT-PCR. As for RNAscope in situ hybridization, Immuno-electron microscopy and behavioral test, adult mice at postnatal 6-12 weeks were used.
Wild animals	No wild animals were used.
Field-collected samples	No field-collected samples were used.
Ethics oversight	All mouse experiments were approved by the Animal Ethics Committee of Shanghai Jiao Tong University School of Medicine (AAALAC accreditation Unit, 001670) and the Institutional Animal Care and Use Committee, the protocol number is A-2018-028.

Note that full information on the approval of the study protocol must also be provided in the manuscript.

Flow Cytometry

Plots

Confirm that:

- [x] The axis labels state the marker and fluorochrome used (e.g. CD4-FITC).
- [x] The axis scales are clearly visible. Include numbers along axes only for bottom left plot of group (a 'group' is an analysis of identical markers).
- [x] All plots are contour plots with outliers or pseudocolor plots.
- [x] A numerical value for number of cells or percentage (with statistics) is provided.

Methodology

Sample preparation	Cells were isolated from Pdgfrα-creER™; Rosa26-mGFP or GAD67-GFP transgenic mice brain, and prepared as described in methods (under Methods section titled NG2 glia and interneurons isolation by FACS).
Instrument	Beckman Coulter Moflo Astrios.

Software	The sorted cells were analyzed with Summit software and collected for smart-seq.
Cell population abundance	About 2~5X10^5 EGFP+ cells were sorted per sample.
Gating strategy	Relevant gating strategies shown in Supplementary Information.

☒ Tick this box to confirm that a figure exemplifying the gating strategy is provided in the Supplementary Information.
