# Supplementary material for: NG2 glia-derived GABA release tunes inhibitory synapses and contributes to stress-induced anxiety
Source: Nat Commun. 2021 Sep 30;12:5740. doi: 10.1038/s41467-021-25956-y (PMC8484468; doi:10.1038/s41467-021-25956-y)
Supplement: Supplementary file 4 — Description of Additional Supplementary Files [file 41467_2021_25956_MOESM4_ESM.pdf]

Title: Supplementary Movie 1

Description: 3D reconstruction of confocal images of NG2 glia and gephyrin-labeled interneurons. The 3D reconstruction of confocal images were simulated with LAS X software from Pdgfrα-creER^{TM}; mGFP mouse hippocampus. The GFP-labeled NG2 glia were visualized with green and gephyrin-labeled inhibitory neurons were labeled with magenta. The image shows a direct somatic contact between one NG2 glia and a gephyrin-labeled interneuron.

Title: Supplementary Movie 2

Description: A primary cultured NG2 cell transfected with VAMP-2-pHuji under TIRFM. The 25 second live images show the fusion events of VAMP-2-pHuji laden vesicles along the processes of one NG2 cell (outlined by yellow dotted lines) obtained from Pdgfrα-creER^{TM};ChR2-eYFP mouse brain before and after blue light stimulation (10 Hz, 60 sec). A substantial increase of VAMP-2-laden vesicles exocytosis after NG2 cell photoactivation can be observed. Frames were acquired by streaming directly to disk at 20 frames per second (50 ms exposure) with the image size of $44.22 \times 36.96\ \mu m^2$. Penetration depth of evanescent field was set to 200 nm. Pixel intensity for the basal control (left) and blue light stimulation (right) were adjusted to the same range by Fiji.

Title: Supplementary Movie 3

Description: Representative time-lapse of Ca^{2+} fluctuation imagings recorded in NG2 glia somata and processes from NG2-creER^{TM};GCaMP6s control (left panels) and CSDS (right panels) mice hippocampal slices with two-photon microscopy. Note the increment of Ca^{2+} fluctuation events occurred mainly in NG2 glia processes in CSDS mice, indicating an intrinsic activation of NG2 glia after mice developed CSDS. The upper panels show the time-lapse of Ca^{2+} imagings as indicated in Fig. 5b, c. The bottom panels are the Ca^{2+} imagings from additional NG2 glia in control and CSDS mice.

Frame size of each movie equals 100 × 100 pixels (0.994 μm per pixel). Scale bar, 20 μm.

Title: Supplementary Movie 4

Description: NG2 glia photostimulation in vivo shows an anxiety-like activity in an open field test. In an open field test chamber, Pdgfrα-creER^{+}; ChR2^{+} mouse (right) shows less time and activity in the center zone compared with that in Pdgfrα-creER^{+}; ChR2^{-} mouse (left) during NG2 glia repetitive photostimulation in a representative 1-min period of activity tracks, which indicates ChR2-expressing NG2 glia activation induces an anxiety-like behavioral activity in an open field test.

Title: Supplementary Movie 5

Description: NG2 glia photostimulation in vivo shows an anxiety-like activity in an elevated plus maze. In an elevated plus maze, Pdgfrα-creER^{+}; ChR2^{+} mouse (right) shows less time and activity in open arms compared with that in Pdgfrα-creER^{+}; ChR2^{-} mouse (left) during NG2 glia repetitive photostimulation in a representative 1-min period of activity tracks, which indicates ChR2-expressing NG2 glia activation enhances an anxiety-like behavioral activity in an elevated plus maze test.
